# Cyclin-dependent kinase 5 mediates pleiotrophin-induced endothelial cell migration

**DOI:** 10.1038/s41598-018-24326-x

**Published:** 2018-04-12

**Authors:** Evgenia Lampropoulou, Ioanna Logoviti, Marina Koutsioumpa, Maria Hatziapostolou, Christos Polytarchou, Spyros S. Skandalis, Ulf Hellman, Manolis Fousteris, Sotirios Nikolaropoulos, Efrosini Choleva, Margarita Lamprou, Angeliki Skoura, Vasileios Megalooikonomou, Evangelia Papadimitriou

**Affiliations:** 1 Laboratory of Molecular Pharmacology, Department of Pharmacy, University of Patras, GR26504 Patras, Greece; 2 Department of Biosciences, School of Science and Technology, Nottingham Trent University, Nottingham, NG11 8NS United Kingdom; 3 Laboratory of Biochemistry, Department of Chemistry, University of Patras, GR26504 Patras, Greece; 4 Ludwig Institute for Cancer Research, Uppsala University, Uppsala, SE-751-05 Sweden; 5 Laboratory of Medicinal Chemistry, Department of Pharmacy, University of Patras, GR26504 Patras, Greece; 6 Computer Engineering and Informatics Department, University of Patras, Patras, Greece; 7 Present Address: Center for Systems Biomedicine, Vatche and Tamar Manoukian Division of Digestive Diseases, David Geffen School of Medicine, University of California at Los Angeles, Los Angeles, CA 90095 USA

## Abstract

Pleiotrophin (PTN) stimulates endothelial cell migration through binding to receptor protein tyrosine phosphatase beta/zeta (RPTPβ/ζ) and α_ν_β_3_ integrin. Screening for proteins that interact with RPTPβ/ζ and potentially regulate PTN signaling, through mass spectrometry analysis, identified cyclin-dependent kinase 5 (CDK5) activator p35 among the proteins displaying high sequence coverage. Interaction of p35 with the serine/threonine kinase CDK5 leads to CDK5 activation, known to be implicated in cell migration. Protein immunoprecipitation and proximity ligation assays verified p35-RPTPβ/ζ interaction and revealed the molecular association of CDK5 and RPTPβ/ζ. In endothelial cells, PTN activates CDK5 in an RPTPβ/ζ- and phosphoinositide 3-kinase (PI3K)-dependent manner. On the other hand, c-Src, α_ν_β_3_ and ERK1/2 do not mediate the PTN-induced CDK5 activation. Pharmacological and genetic inhibition of CDK5 abolished PTN-induced endothelial cell migration, suggesting that CDK5 mediates PTN stimulatory effect. A new pyrrolo[2,3-*α*]carbazole derivative previously identified as a CDK1 inhibitor, was found to suppress CDK5 activity and eliminate PTN stimulatory effect on cell migration, warranting its further evaluation as a new CDK5 inhibitor. Collectively, our data reveal that CDK5 is activated by PTN, in an RPTPβ/ζ-dependent manner, regulates PTN-induced cell migration and is an attractive target for the inhibition of PTN pro-angiogenic properties.

## Introduction

Pleiotrophin (PTN) is a secreted growth factor that binds to receptor protein tyrosine phosphatase beta/zeta (RPTPβ/ζ) and α_ν_β_3_ integrin to stimulate human endothelial cell migration^1–3^. PTN regulates angiogenesis directly, through stimulation of endothelial cells, and indirectly through its regulatory role on the angiogenic effects of vascular endothelial growth factor A^3^. RPTPβ/ζ initiates PTN signalling through cellular Src kinase (c-Src) dephosphorylation and activation that consequently leads to β_3_ Tyr773 phosphorylation and activation of phosphatidylinositol 3-kinase (PI3K)^2,4^. Moreover, other signalling molecules, such as focal adhesion and ERK1/2 kinases^1^, nitric oxide^5^ and xanthine oxidase^6^, have been shown to be activated down-stream of RPTPβ/ζ and required for PTN-induced endothelial cell migration. However, the cross-talk among all the identified signalling molecules involved, as well as novel unknown molecules that may mediate the RPTPβ/ζ migratory signalling pathways, are still to be investigated.

Cyclin-dependent kinases constitute a family of small serine-threonine kinases known for their major role in the progression of cell cycle. Among the members of the family, cyclin-dependent kinase 5 (CDK5) has no effect on cell cycle regulation and was initially reported as a neuronal kinase, expressed solely in the nervous system^7^. CDK5 is activated through binding to specific protein partners, p35, p39 and cyclin I, and its activity is determined by the amount of available binding partners^8,9^. Nowadays, it is known to be expressed in several extra neuronal cell types and tissues and has several pathophysiological roles, among which regulation of angiogenesis and cancer growth^8^. CDK5 has been recently implicated in the development and progression of a plethora of cancer types (including hepatocellular carcinoma, head and neck squamous carcinoma, thyroid, breast and prostate cancer) and in important processes, such as angiogenesis^9–13^ and lymphangiogenesis^14^. At the cellular level, a major role of CDK5 is to control actin remodelling^15^, a process closely linked to cell migration and angiogenesis. However, it remains unclear how CDK5 is activated during angiogenesis.

In the current work, mechanistic and functional studies were performed to elucidate in depth the molecular players involved in PTN-induced, RPTPβ/ζ-mediated, cell migration with an emphasis on CDK5.

## Results

### RPTPβ/ζ directly interacts with CDK5 and its activator p35

**Figure 1:**
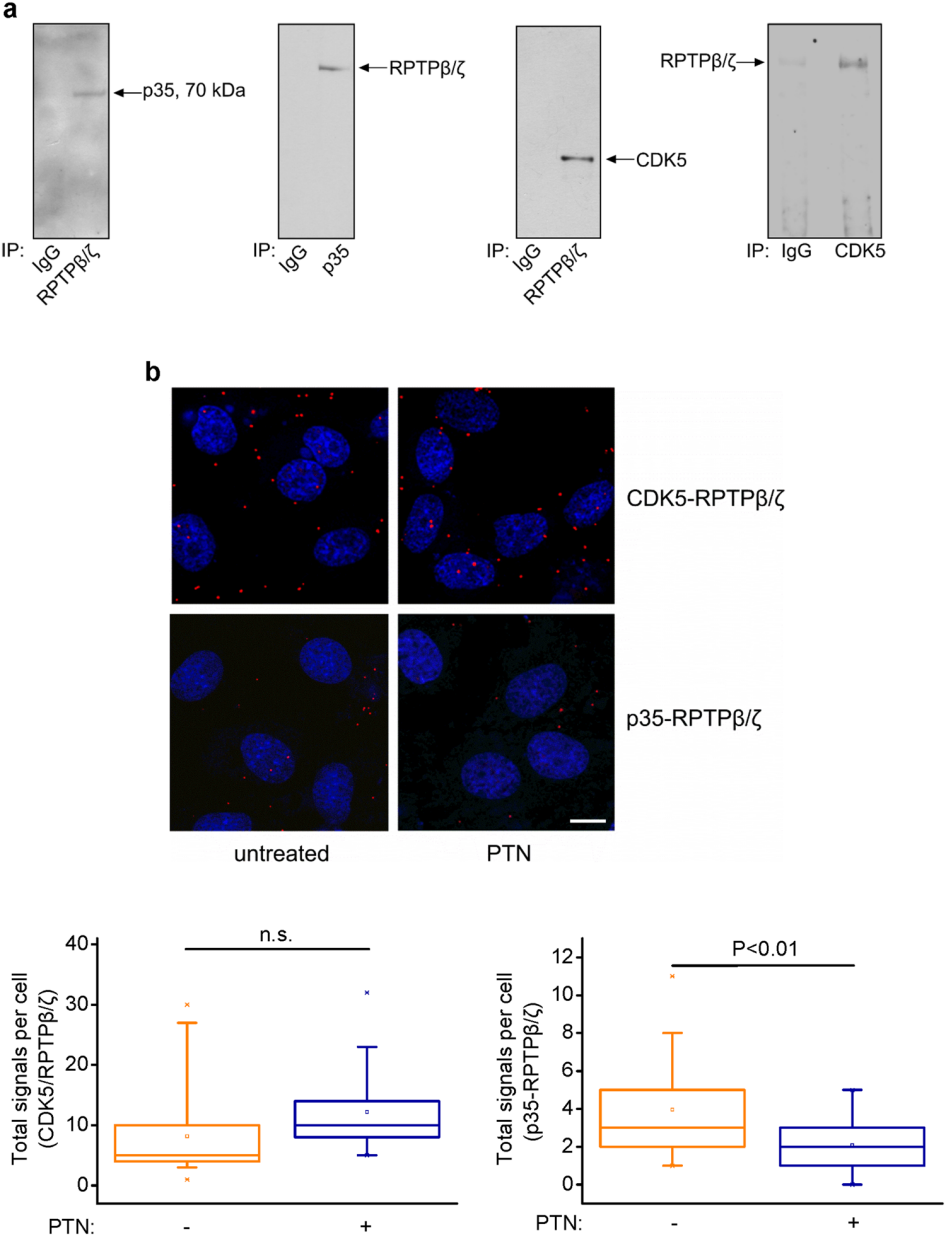
CDK5 and its activator p35 interact with RPTPβ/ζ. (**a**) HUVEC lysates were immunoprecipitated with an antibody for RPTPβ/ζ, p35 or CDK5 and analysed by Western blot for the presence of p35 and CDK5 or RPTPβ/ζ. IgG was used as a negative control. Pictures are representative from four independent experiments. (**b**) Formation of CDK5-RPTPβ/ζ and p35-RPTPβ/ζ complexes as evidenced by *in situ* PLA in HUVEC in the absence or presence of exogenous PTN (100 ng/ml) for 10 min. Red color indicates the studied complexes and blue corresponds to nuclear Draq5 staining. Pictures are representative from two independent experiments. Scale bar corresponds to 10 μm. The box plots indicate the median and range of the detected signals from three independent experiments. n > 20 image fields, with ~4 cells per image per sample type. Each sample run at least in duplicate.

To explore RPTPβ/ζ binding partners that potentially contribute to PTN-induced signaling, human umbilical vein endothelial cell (HUVEC) lysates were immunoprecipitated with an anti-RPTPβ/ζ antibody and the co-immunoprecipitated proteins were identified through MALDI-TOF MS analysis. Among the proteins co-immunoprecipitated with RPTPβ/ζ, a protein band of about 70 kDa was found to contain peptides identical to p35 (6 matched peptides; minimum sequence coverage 25%, accession No: NP_003876, Table 1). This interaction was verified by Western blot analysis (Fig. 1a), whereas *in situ* proximity ligation (PLA) assays (Fig. 1b) demonstrated formation of direct RPTPβ/ζ-p35 complexes. Interestingly, from both the mass spectrometry and the Western blot assays, it was found that RPTPβ/ζ co-immunoprecipitates with a protein identified as p35 and recognized by a p35-specific antibody, respectively, which appears as a ~70 kDa p35 dimer (Fig. 1a). CDK5 was also found to co-immunoprecipitate (Fig. 1a) and interact (Fig. 1b) with RPTPβ/ζ, identifying the latter as a novel binding partner of CDK5/p35. CDK5-RPTPβ/ζ interaction does not seem to be affected, while p35-RPTPβ/ζ interaction was decreased 10 min after HUVEC stimulation with PTN, as shown by the *in situ* PLA assays (Fig. 1b).

**Table 1:** Identification of cyclin-dependent kinase 5 activator 1, p35 (alt name: cyclin-dependent kinase 5 regulatory subunit 1) by peptide mass fingerprint analysis (IP: anti-RPTPβ/ζ).

Measured Mass (M)	Computed Mass	Error (Da)	Residues Start	Residues To	Missed Cut	Peptide Sequence
1193.617	1193.614	0.003	261	271	0	CLSVINLMSSK
1209.611	1209.609	0.002	261	271	0	CLSVINLMSSK
1218.613	1218.635	−0.022	128	140	0	APHPAVSSAGTPK
1309.671	1309.669	0.002	1	12	0	MGTVLSLSPSYR
2282.130	2282.103	0.027	272	290	0	MLQINADPHYFTQVFSDLK
2307.129	2307.148	−0.020	13	34	1	KATLFEDGAATVGHYTAVQNSK

This table shows the 6 tryptic peptides identified, expanding throughout the 25% of the human p35 sequence.

### CDK5 is required for PTN-induced cell migration

**Figure 2:**
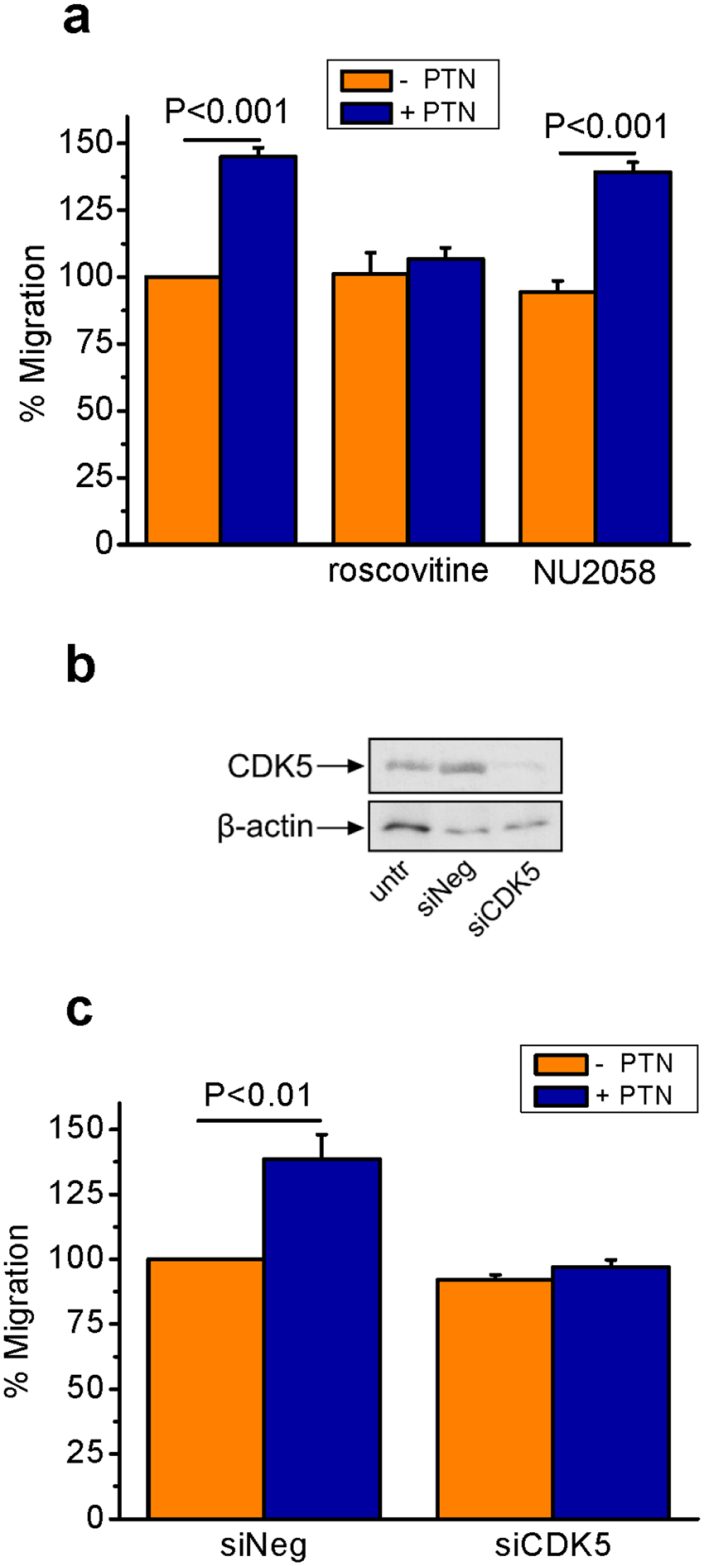
CDK5 is involved in PTN-induced cell migration. (**a**) Serum-starved HUVEC were stimulated with PTN (100 ng/ml) in the absence or presence of roscovitine (10 μΜ) or NU2058 (10 μΜ). Migration was studied using the transwell assay, as described in Materials and Methods. Results are expressed as mean ± SE (n = 4) of the percentage change compared to untreated cells (set as default 100%). (**b**) Representative picture from Western blot analysis of total cell lysates following downregulation of CDK5 by siRNA (50 nM) in HUVEC. Beta-actin was used as the loading control. (**c**) Following downregulation of CDK5, serum-starved HUVEC were stimulated with PTN (100 ng/ml) and migration was measured using the transwell assay. Results are expressed as mean ± SE (n = 3) of the percentage change compared to untreated siNeg cells (set as default 100%). Untr, untransfected cells; siNeg, cells transfected with a negative control siRNA; siCDK5, cells transfected with siRNA for CDK5. F values of the ANOVA tests are 22.5 for (**a**) and 17.4 for (**c**).

To investigate whether CDK5 has a role in PTN-induced endothelial cell migration, the effect of roscovitine (a CDK 1, 2 and 5 inhibitor) and NU2058 (a CDK 1 and 2 inhibitor) was tested. As shown in Fig. 2a, PTN-induced HUVEC migration was abolished in the presence of roscovitine but not NU2058, suggesting a CDK5 specific effect. The role of CDK5 in PTN-induced migration was verified through CDK5 suppression by means of siRNA (Fig. 2b). CDK5 knockdown results in significant inhibition of PTN-induced HUVEC migration (Fig. 2c). Similarly, pharmacological CDK5 inhibition by roscovitine or genetic CDK5 down-regulation, by means of siRNA, abolished PTN-induced migration of human glioma U87MG cells (Supplementary Fig. S1).

### PTN enhances CDK5 activity

**Figure 3:**
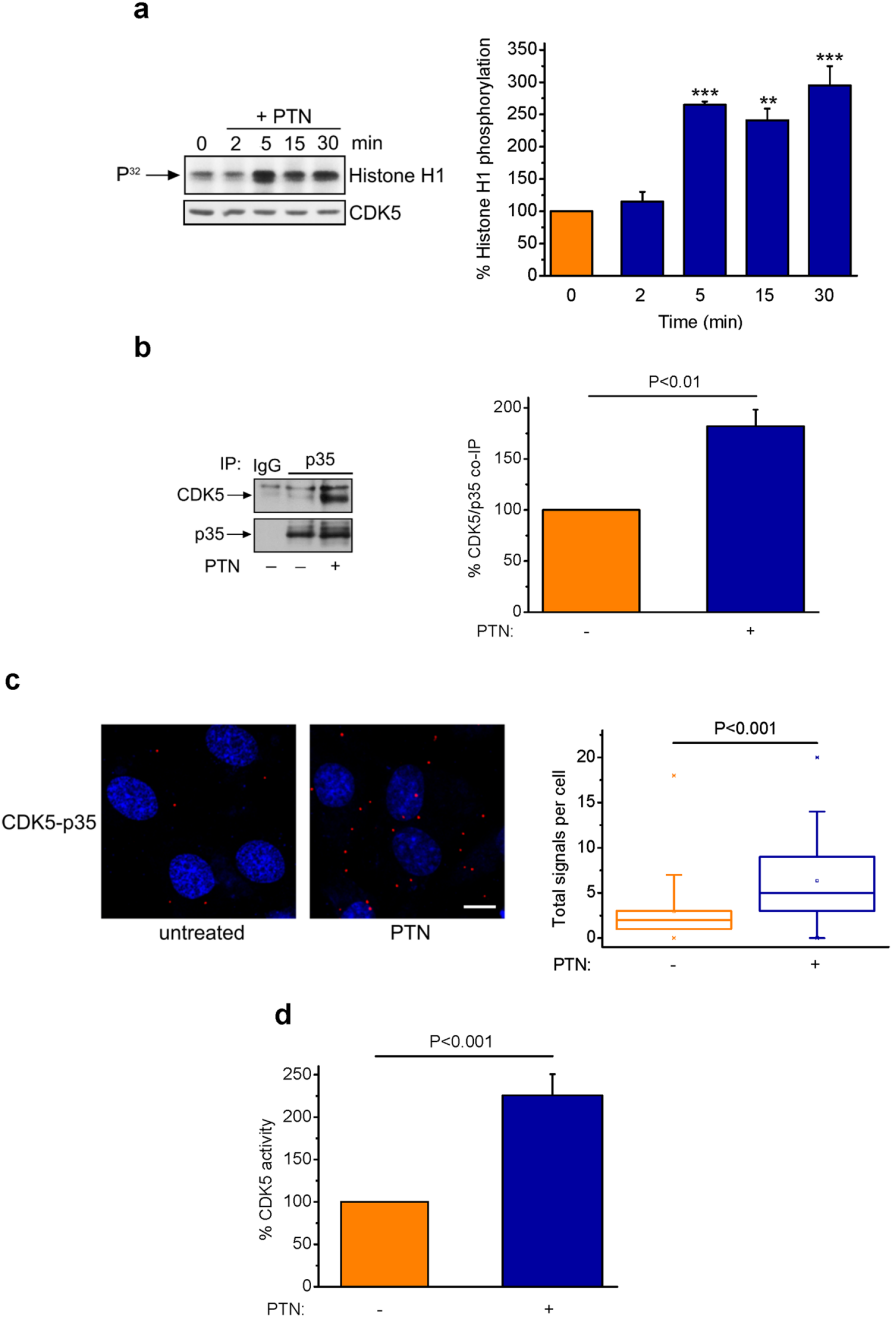
PTN enhances CDK5 activity. (**a**) Effect of PTN (100 ng/ml) on CDK5 activity as assessed by histone H1 phosphorylation *in vitro*. Total HUVEC lysates were immunoprecipitated with a CDK5 antibody and immunoprecipitates were incubated with histone Η1 (20 μg) and [γ-^32^P] ATP (20 μCi) for 30 min at 30 °C, as described in Materials and Methods. A representative autoradiography from three independent experiments is presented. The bands corresponding to phosphorylated histone Η1 were quantified by image analysis software and normalized against total CDK5 levels in each sample. Results are expressed as mean ± SE (n = 3) of the percent change of histone H1 phosphorylation compared to untreated cells (set as default 100%). Asterisks denote statistically significant differences from the untreated cells; **P < 0.01, ***P < 0.001. F value of the ANOVA test is 27.2. (**b**) HUVEC lysates were immunoprecipitated with a p35 antibody and the immunoprecipitates were analysed by Western blot for the presence of CDK5 and p35. CDK5 and p35 protein amounts were quantified and the ratio of p35 to CDK5 was calculated in each lane. Results are expressed as mean ± SE (n = 8) of the percent change of CDK5/p35 ratio in PTN-stimulated vs. the untreated cells (set as default 100%). (**c**) Formation of CDK5-p35 complexes, as evidenced by *in situ* PLA in HUVEC in the absence or presence of exogenous PTN (100 ng/ml). Scale bar corresponds to 10 μm. Red color indicates the studied complexes and blue corresponds to nuclear Draq5 staining. The box plots indicate the median, mean and range of the detected signals from three independent experiments. n > 20 image fields, with ~4 cells per image per sample type. Each sample run at least in duplicate. (**d**) CDK5 activity was measured by using the ADP-Glo Kinase Assay, in CDK5 immunoprecipitates from HUVEC. Results are expressed as mean ± SE (n = 14) of the percent change in CDK5 activity in PTN-stimulated vs the untreated cells (set as default 100%).

Given that CDK5 interacts with RPTPβ/ζ and is involved in PTN-induced cell migration, we further investigated whether PTN affects CDK5 activity. To this end, HUVEC total cell lysates were immunoprecipitated with an anti-CDK5 antibody and *in vitro* Histone H1 phosphorylation assays were employed. Maximum CDK5 activity was observed within 5 min, following PTN stimulation, and was sustained for up to 30 min. Total CDK5 was used as the loading control (Fig. 3a). Considering that the CDK5/p35 interaction leads to CDK5 activation^16^, we additionally tested the effect of PTN on CDK5/p35 interaction, as a means of CDK5 activation. Cells treated with PTN for 10 min were lysed, immunoprecipitated with a p35 antibody and analyzed by Western blot for CDK5. As shown in Fig. 3b, PTN induced CDK5/p35 interaction, in line with increased CDK5 activity. Increased CDK5/p35 interaction was verified by PLA assays (Fig. 3c), as well as non-radioactive CDK5 activity assay (described in Materials and Methods) (Fig. 3d).

### RPTPβ/ζ and PI3K are necessary for the PTN-induced CDK5 activation

**Figure 4:**
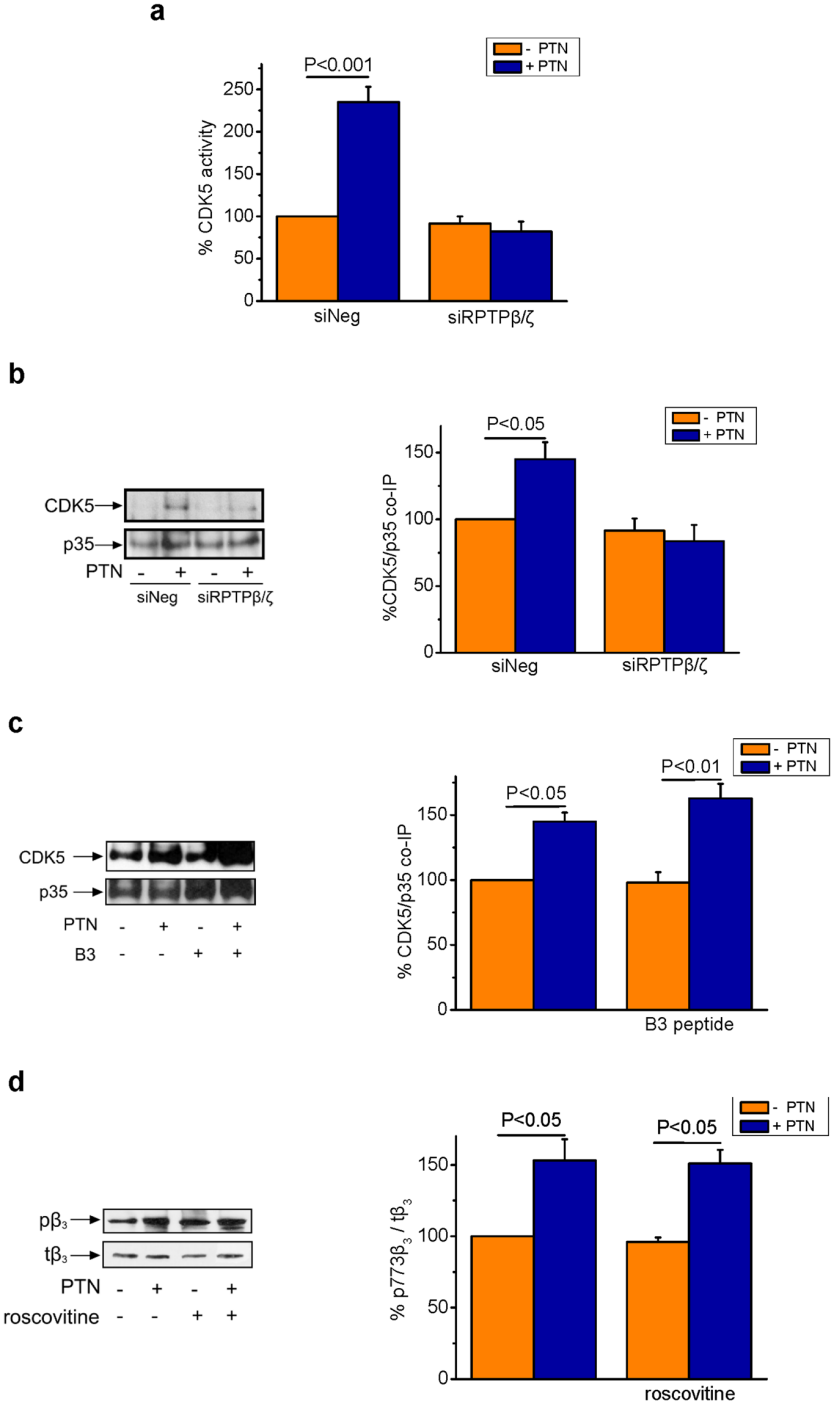
PTN-induced CDK5 activation depends on RPTPβ/ζ but not α_ν_β3 integrin. (**a**) CDK5 activity was measured using the ADP-Glo Kinase Assay, in CDK5 immunoprecipitates from HUVEC following RPTPβ/ζ knockdown through siRNA (50 nM). Results are expressed as mean ± SE (n = 3) of the percent change in CDK5 activity in PTN-stimulated vs the untreated siNeg cells (set as default 100%). (**b**) HUVEC lysates following RPTPβ/ζ knockdown, through siRNA, were immunoprecipitated with a p35 antibody and the immunoprecipitates were analysed by Western blot for the presence of CDK5 and p35. CDK5 and p35 protein amounts were quantified and the ratio of p35 to CDK5 was calculated in each lane. Results are expressed as mean ± SE (n = 3) of the percent change of CDK5/p35 ratio in PTN-stimulated vs. the untreated siNeg cells (set as default 100%). siNeg, cells transfected with a negative control siRNA; siRPTPβ/ζ, cells transfected with siRNA for RPTPβ/ζ. (**c**) HUVEC cells were treated in the presence or absence of peptide B3 (1 μg/ml), known to block PTN-α_ν_β_3_ interaction. Whole cell lysates were immunoprecipitated for p35 and the immunoprecipitates were analysed by Western blot for the presence of CDK5 and p35. CDK5 and p35 protein amounts were quantified and the ratio of p35 to CDK5 was calculated in each lane. Results are expressed as mean ± SE (n = 3) of the percent change of CDK5/p35 ratio in stimulated vs. the untreated cells (set as default 100%). (**d**) HUVEC were incubated with PTN (100 ng/ml) in the absence or presence of roscovitine (10 μΜ). Phosphorylation of β_3_Tyr773 was estimated in total cell lysates by Western blot as described in Materials and Methods. Phospho-β_3_Tyr773 (pβ_3_) and total β_3_ (tβ_3_) amounts were quantified and the ratio pβ_3_/tβ_3_ was calculated in each lane. Results are expressed as mean ± SE (n = 3) of the percent change in phospho-β_3_Tyr773 relative amounts in PTN-stimulated vs. the untreated cells (set as default 100%). F values of the ANOVA tests are 38.7 for (**a**), 7.8 for (**b**), 18.2 for (**c**) and 11.9 for (**d**).

Since CDK5 interacts with RPTPβ/ζ and the latter is required for PTN-induced migratory signaling^1,3^, we tested whether RPTPβ/ζ is involved in PTN-induced CDK5 activation. To this end, RPTPβ/ζ was down-regulated by means of siRNA, as previously described^1^ and CDK5 activity or its interaction with p35 were assessed (with or without PTN stimulation). As shown in Fig. 4a and b, RPTPβ/ζ is required for PTN-induced CDK5 activation and interaction with p35.

PTN-induced cell migration is also mediated through α_ν_β_3_ integrin and specifically the 177-184 cysteine loop of the β_3_ extracellular domain^2^. To investigate whether PTN induces CDK5 activation through α_ν_β_3_, we used a synthetic peptide ^177^CYDMKTTC^184^ (B3 peptide) that corresponds to the cysteine loop mentioned above. This exogenously added peptide acts as a decoy for PTN thus inhibiting its interaction with α_v_β_3_^2^. Peptide B3 had no effect on PTN-induced CDK5-p35 interaction (Fig. 4c), suggesting that PTN binding to α_v_β_3_ integrin is not involved in PTN-induced CDK5 activation. In the same line, PTN increases CDK5 activity and interaction with p35, in an RPTPβ/ζ-mediated manner, in rat glioma C6 cells that do not express α_ν_β_3_^2,4^. Interestingly, in these cells, p35 and CDK5 co-immunoprecipitate with RPTPβ/ζ (Supplementary Fig. S2). Roscovitine did not inhibit PTN-induced β_3_Tyr773 phosphorylation either (Fig. 4d), suggesting that CDK5 is not upstream of α_ν_β_3_.

**Figure 5:**
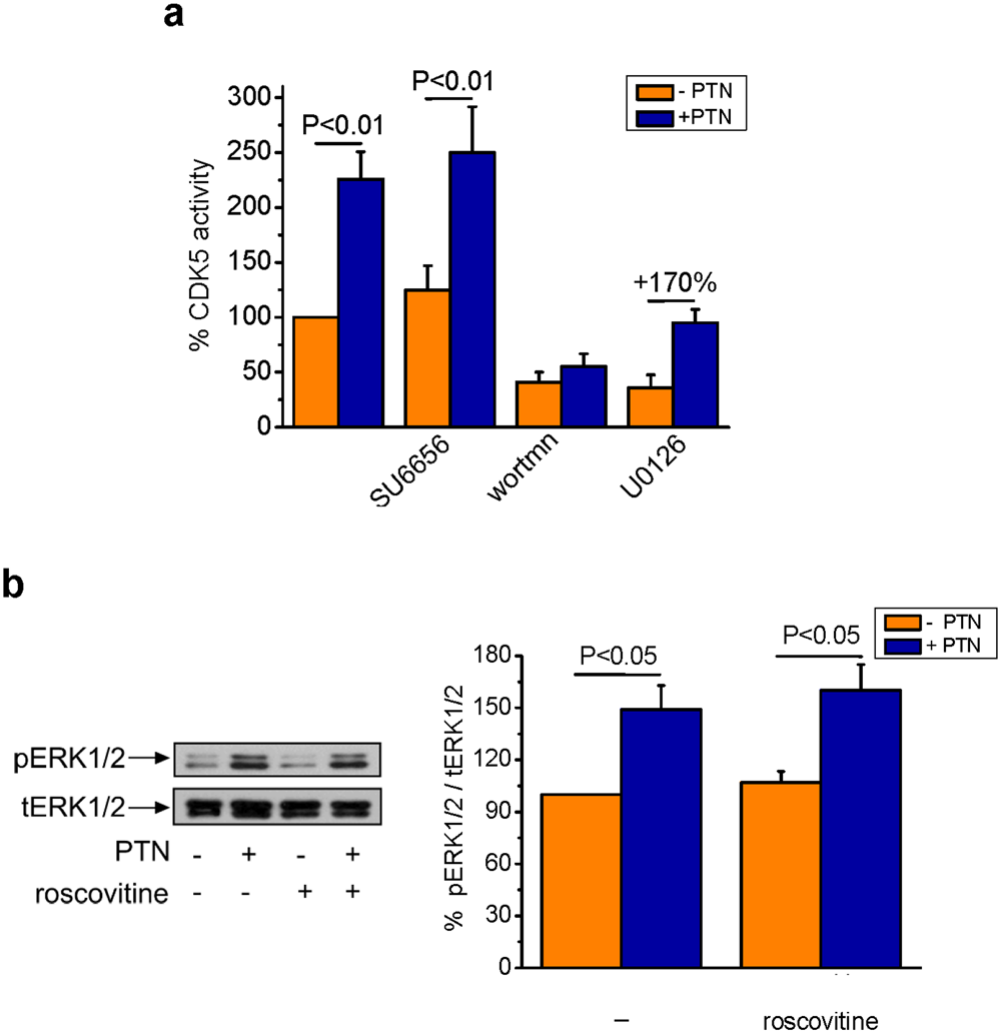
PTN-induced CDK5 activation depends on PI3K but not c-Src or ERK1/2. (**a**) CDK5 activity was measured in CDK5 immunoprecipitates from HUVEC previously treated with the following pharmacological inhibitors: SU6656 (10 μΜ), wortmannin (wortmn, 100 nM) or U0126 (20 nM). CDK5 activity was evaluated by using the ADP-Glo Kinase Assay. Results are expressed as mean ± SE (n = 3) of the percent change in CDK5 activity in PTN-stimulated vs the untreated cells (set as default 100%). (**b**) HUVEC were incubated with PTN (100 ng/ml) in the absence or presence of roscovitine (10 μΜ). Phosphorylation of ERK1/2 was estimated in total cell lysates by Western blot as described in Materials and Methods. Phospho-ERK1/2 (pERK1/2) and total ERK1/2 (tERK1/2) amounts were quantified and the ratio pERK1/2/tERK1/2 was calculated in each lane. Results are expressed as mean ± SE (n = 3) of the percent change in phospho-ERK1/2 relative amounts in PTN-stimulated vs. the untreated cells (set as default 100%). F values of the ANOVA tests are 15.8 for (**a**) and 7.8 for (**b**).

To further characterize the PTN-induced signaling pathway that activates CDK5, downstream of RPTPβ/ζ, we used pharmacological inhibitors of c-Src, PI3K and ERK1/2 kinases, known to act downstream of RPTPβ/ζ and to affect PTN-induced endothelial cell migration^1^. Inhibition of c-Src by SU6656 (a c-Src family kinase, SFK, inhibitor) or ERK1/2 by the MEK inhibitor U0126, did not affect PTN-induced CDK5 activation, suggesting that PTN activates CDK5 in an SFK- and ERK1/2-independent manner. On the contrary, the broad spectrum PI3K inhibitor wortmannin abolished PTN-induced CDK5 activation, suggesting that PI3K lies upstream of CDK5 and is necessary for its activation (Fig. 5a). Inhibition of CDK5 activity by roscovitine did not affect PTN-induced ERK1/2 activation either (Fig. 5b), suggesting that CDK5 is not upstream of ERK1/2.

One other important signaling molecule that has been shown to be tyrosine phosphorylated by ΡΤΝ and to translocate into the nucleus downstream of RPTPβ/ζ, is β-catenin^17^. Stimulation of HUVEC with PTN for 10 min, did not affect β-catenin tyrosine phosphorylation or β-catenin cell membrane localization (Supplementary Fig. S3), suggesting that β-catenin is not involved in this pathway in endothelial cells.

### Rac1 participates in the PTN migratory signaling pathway without affecting CDK5 activation

Based on a previous study, Cdk5 does not influence focal adhesion dynamics and microtubule organization in HUVEC but affects actin cytoskeleton and Rac1 activation^9^. It is also known that integrin-mediated cell migration involves Rac1^18^. In the present work, to further elucidate how CDK5 and α_ν_β_3_ may cross-talk to mediate PTN-induced endothelial cell migration, we studied a possible involvement of the GTPase Rac1. To this end, we used a specific Rac1 inhibitor, NSC23766, which can block the effect of Rac1 without interfering with the effects of RhoA and Cdc42^19^. As shown in Supplementary Fig. S4, NSC23766 abolished the stimulatory effect of PTN on HUVEC migration, suggesting that Rac1 is involved in the PTN migratory signaling pathway. NSC23766 did not affect PTN-induced CDK5 activation, suggesting that Rac1 is not upstream of CDK5 in the studied PTN signaling pathway.

### Effect of pyrrolo[2,3-*α*]carbazole derivatives on PTN-induced CDK5 activation and endothelial cell migration

**Figure 6:**
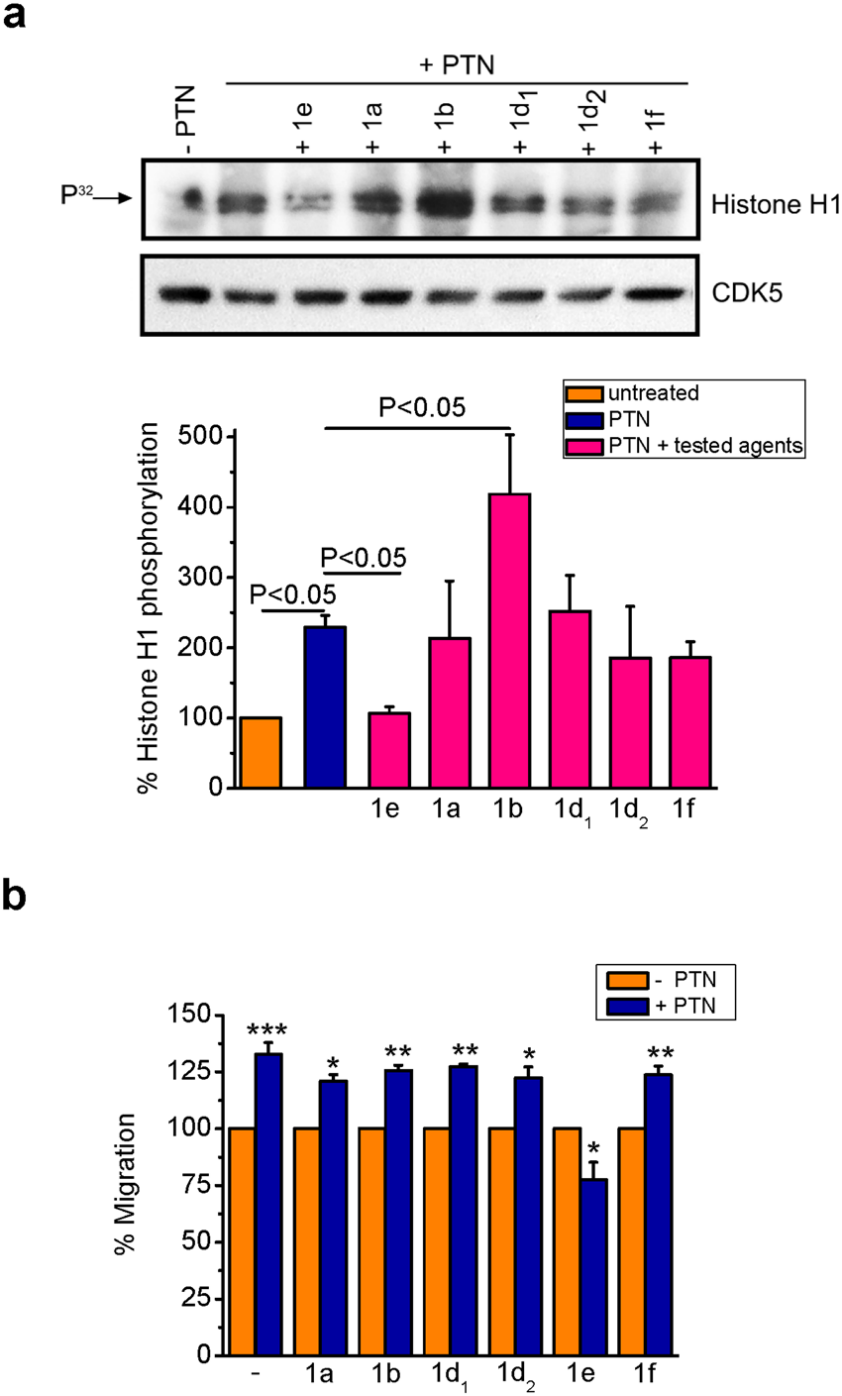
Effect of the studied pyrrolo[2,3-α]carbazole derivatives on PTN-induced CDK5 activation and cell migration *in vitro*. (**a**) HUVEC were treated with the tested agents (10 μΜ) for 30 min, prior to stimulation with PTN (100 ng/ml) for 5 min. Whole cell lysates were immunoprecipitated for CDK5 and immunoprecipitates were incubated with histone Η1 (20 μg) and [γ-^32^P] ATP (20 μCi) for 30 min at 30 °C, as described in Materials and Methods. A representative autoradiography from three independent experiments is presented. The bands corresponding to phosphorylated histone Η1 were quantified by image analysis software and were normalized against total CDK5 levels in each sample. Results are expressed as mean ± SE (n = 3) of the percent change of histone H1 phosphorylation compared to untreated cells (set as default 100%). (**b**) Serum-starved HUVEC were stimulated by PTN (100 ng/ml) in the absence or presence of the tested agents (10 μΜ). Migration was measured using the transwell assay, as described in Materials and Methods. Results are expressed as mean ± SE (n = 3) of the percentage change compared to the corresponding untreated cells (set as default 100%). Asterisks denote statistically significant differences from the untreated cells; *P < 0.05, **P < 0.01, ***P < 0.001. F values of the ANOVA tests are 6.6 for (**a**) and 19.3 for (**b**).

We have previously described the synthesis of pyrrolo[2,3-*α*]carbazole derivatives and reported their *in vitro* effect on cyclin dependent kinase 1 (CDK1)^20^ and topoisomerase I^21^ activity and endothelial cell proliferation^21^. The aim of the present work was to study the effect of the same pyrrolo[2,3-*α*]carbazole derivatives (Supplementary Fig. S5) on PTN-induced CDK5 activation and migration in HUVEC. Among all compounds tested, only the compound 1e exhibits a significant suppressive effect on PTN-induced CDK5 activation (Fig. 6a) and PTN-induced HUVEC migration (Fig. 6b). Similarly, compound 1e abolished PTN-induced migration of U87MG glioma cells (Supplementary Fig. S6).

### CDK5 expression and activity are developmentally regulated in the chicken embryo chorioallantoic membrane

The chicken embryo chorioallantoic membrane (CAM) is a widely used *in vivo* angiogenesis assay^22^ and PTN has been shown to play a significant role in angiogenesis in this tissue^23^. In the present work, we studied CDK5 expression and activation during the development of CAM. As shown in Supplementary Fig. S7, expression and activation of CDK5 are significantly increased at day 9, compared with day 6, of embryo development coinciding with increased tissue angiogenesis^24^. Levels of both expression and activity remain practically constant till day 15 of embryo development and significantly decrease at day 18.

## Discussion

In the current study we tried to characterize the endothelial cell signaling cascade involved in the pro-migratory effect of PTN. We mainly focused on CDK5 and its activator p35 based on the initial observation that p35 interacts with RPTPβ/ζ, the receptor through which PTN initiates the endothelial migratory signaling^1,2^. Interestingly, we observed that RPTPβ/ζ mainly interacts with a ~70 kDa protein identified by MALDI-TOF and Western blot analyses as p35, which is possibly a p35 dimer. The literature on the existence of p35 dimers is very limited, with only one study showing that p35 homodimerizes and induces F-actin bundles formation^25^. PTN has been shown to differentially affect actin cytoskeleton in HUVEC^26^. Through specific pharmacological inhibition, we were able to show that Rac1 activation, a major player in actin cytoskeleton rearrangements, is involved in PTN-induced migratory signaling. Whether and how RPTPβ/ζ is involved in Rac1 activation and consequently to actin filament rearrangements, is of great interest and a mechanism to be interrogated in future studies.

Although CDK5 is mostly referred to as neuronal kinase, a possible role in angiogenesis has been further attributed to this molecule. The first report showed that CDK5 is expressed in proliferating and not quiescent bovine aortic endothelial cells; its expression is increased by basic fibroblast growth factor and mediates cell proliferation^27^. CDK5 regulates angiogenesis *in vitro* and *in vivo* in different settings^9,12,28^ and several roscovitine analogues elicit antiangiogenic effects^7,29–31^. This is the first study functionally linking CDK5 with PTN signaling. Previous studies indicate similarities between these two molecules in normal and pathological conditions, especially in the nervous system, where they are both over-expressed^32,33^. Both PTN^34,35^ and CDK5^36,37^ are significant in brain growth and development through induction of neurite formation. PTN suppresses Long Term Potentiation (LTP) in hippocampus and plays a role in the regulation of learning-related behavior^32,38^. CDK5 activation is implicated in LTP inhibition^39^, while conditional knockout CDK5 mice exhibit increased LTP^40^. CDK5 and p35/p25 may play a role in the pathogenesis of Alzheimer’s disease(AD) leading to abnormal phosphorylation of substrates such as tau^15^. ΡΤΝ is overexpressed in the brains of patients with AD^41,42^, but its function remains unknown. Both PTN and CDK5 play important role in neuromuscular junction via their participation in the accumulation of acetylcholine receptors^43,44^. Finally, PTN is overexpressed in microvessels after acute ischemic brain injury^45^, while CDK5 shows increased expression in endothelial cells after ischemic brain injury^46^. The latter aligns with our results showing that CDK5 mediates PΤΝ-induced endothelial cell migration.

Our previous studies have reported integrin α_ν_β_3_ as a key regulator of PTN-induced endothelial cell migration through the formation of a functional complex with RPTPβ/ζ^2^. In the present study, we found that α_ν_β_3_ is not involved in PTN-induced CDK5 activation and CDK5 is not upstream of β_3_Tyr773 phosphorylation. This suggests that CDK5 activation is part of an RPTPβ/ζ-mediated signalling pathway independent of α_ν_β_3_. However, the data showing that both α_ν_β_3_^2^ and CDK5 (present study) are required for PTN-induced endothelial cell migration, indicate a possible cross-talk between the two pathways. One possibility would be through Rac1 activation downstream of CDK5. CDK5-mediated Rac1 activation in HUVEC has been previously shown to be significant for endothelial cell migration^9^ and this agrees with our data showing that Rac1 mediates PTN-induced migration, without being upstream of CDK5. Rac1 has been previously shown to be activated in endothelial cells and mediate cell migration, downstream of PI3K but independently of Akt and ERK1/2^47^. Similarly, our data show that PTN does not activate Akt in endothelial cells^1^ and PTN-induced CDK5 and ERK1/2 activation are independent of each other (this study). Interestingly, Rac1 in *Drosophila* regulates the proper cellular localization of β-integrin Myospheroid^48^, and in mammalian cells integrin recycling and cell migration involve Rac1^18^. It is tempting therefore to speculate that PTN through CDK5 activates Rac1, which then controls recycling of α_ν_β_3_ and thus, endothelial cell migration. Involvement of CDK5 in α_ν_β_3_-mediated cell migration is further supported by the observation that in C6 glioma cells, that do not express α_ν_β_3_^2^, pharmacological inhibition of CDK5 by roscovitine has no effect in the inhibitory effect of PTN in the migration of these cells (Supplementary Fig. S2d).

To further elucidate the signaling pathway that participates in PTN-induced CDK5 activation, we used pharmacological inhibitors of kinases that had been previously found to regulate PTN-induced endothelial cell migration down-stream of RPTPβ/ζ. The specific c-Src inhibitor SU6665 did not affect PTN-induced CDK5 activation. Although c-Src interacts with RPTPβ/ζ and is activated by PTN, down-stream of RPTPβ/ζ and upstream of α_ν_β_3_^1,2^, it is not involved in CDK5 activation by PTN. SU6656 inhibits all SFK members, suggesting that Fyn that has been also shown to be activated by PTN downstream of RPTPβ/ζ^49^ is not involved in CDK5 activation either. In contrast to other cell types, such as glioma U373 cells, 3T3-L1 preadipocytes, fetal alveolar epithelial type II cells and cultured embryonic mouse (E14.5) neurons^3^, PTN stimulation of HUVEC had no effect on β-catenin tyrosine phosphorylation or cell membrane localization, suggesting that β-catenin is not implicated in the PTN pathway that leads to CDK5 activation. The reasons for the observed discrepancy related to β-catenin activation between HUVEC and the other types of cells are not known but could be due to the different set of receptors or co-receptors that participate in the effect of PTN in each cell type, or to the different ways of RPTPβ/ζ signaling initiation. It has been previously shown that PTN induces RPTPβ/ζ dimerization that results in inhibition of tyrosine phosphatase activity and increased tyrosine phosphorylation of several substrates, such as Fyn and β-catenin^3,17,49^. On the other hand, in HUVEC, we have previously shown that upon PTN binding, RPTPβ/ζ dephosphorylates Tyr527 of c-Src leading to its activation, subsequent tyrosine phosphorylation of α_ν_β_3_ and finally increased endothelial cell migration^2,3^. The differences between the two possible modes of signaling downstream of RPTPβ/ζ are being investigated.

Similarly to SFKs, PTN-induced ERK1/2 activation is unrelated to CDK5 activation. On the other hand, PI3K was found to be upstream of CDK5 and affect both basal and PTN-induced CDK5 activity. These data are in line with previous observations that PI3K activation by brain-derived neurotrophin factor induces CDK5/p35 interaction and CDK5 activity^50^, whereas PI3K inhibition leads to decreased CDK5/p35 activity^51^. This study may contradict our previous findings that PI3K lies down-stream of α_ν_β_3_^4^. However, in all cases, we have used wortmannin a non-isoform specific PI3K inhibitor, which may inhibit different isoforms that function down-stream or upstream of α_ν_β_3_. Alternatively, the same PI3K isoform might participate in two parallel signaling pathways that are both downstream of RPTPβ/ζ.

Among a series of pyrrolo[2,3-*α*]carbazoles, we have identified one compound (1e) that inhibits CDK1 activity^20^, while the rest of them have a significant topoisomerase Ι inhibitory activity *in vitro*^21^. Besides topoisomerase I, compound 1e had no effect on the tyrosine kinase activity of several growth factor receptors^20^, suggesting selectivity for CDK1 and potentially other CDKs. In the present study, we show that 1e inhibits CDK5, which may be attributed to the high sequence homology of CDK5 with the mitotic CDK1^52^ and as with previously developed inhibitors, including roscovitine, it inhibits both CDKs. The fact that compound 1e completely inhibited PTN-induced endothelial cell migration, further supports a central role for CDK5 in the effects of PTN on cell motility.

The CDK5 inhibitor roscovitine abolishes vascular endothelial growth factor-induced angiogenesis in the chicken embryo CAM^9^. In the present study, we show that CDK5 expression and activation, in chicken embryo CAM, is significantly increased between days 6 and 9 of embryo development, when the highest rate of endothelial cell proliferation is observed in this tissue^24^. High levels of activity remain till maturation of the tissue blood vessels (day 15)^24^ and are decreased at later developmental stages. The levels of CDK5 activity do not correlate perfectly with those of ΡΤΝ or RPTPβ/ζ in the same tissue, which seem to decrease after day 12 of embryo development^23^. This indicates that CDK5 may be also regulated by other growth factors involved in CAM angiogenesis^22^, such as bFGF^27^.

**Figure 7:**
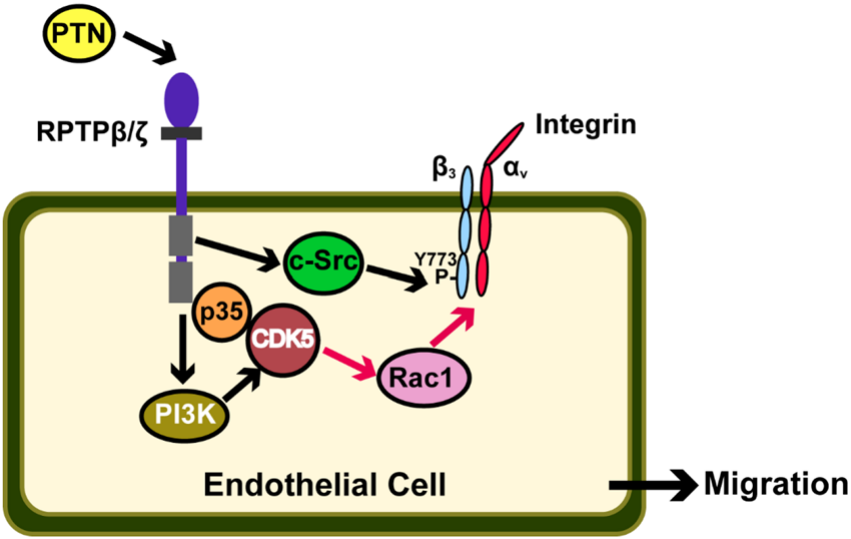
Schematic representation of the proposed PTN pro-migratory signaling pathway that leads to CDK5 activation in human endothelial cells. PTN induces RPTPβ/ζ stimulation that triggers an RPTPβ/ζ-p35-CDK5 interaction. This event leads to CDK5 activation and is independent of the PTN-induced migratory signals through integrin α_ν_β_3_. Furthermore, RPTPβ/ζ stimulation may lead to CDK5 activation through a PI3K-dependent manner. Finally, we show that Rac1 lies downstream of CDK5 and is necessary for PTN-induced endothelial cell migration.

In summary, in the present study we have identified an interaction between RPTPβ/ζ, p35 and CDK5. We showed that PTN induces CDK5/p35 interaction and CDK5 activation downstream of RPTPβ/ζ, and that CDK5 activation is indispensable for PTN-induced migration of endothelial cells (Fig. 7).

## Materials and Methods

### Materials

Human recombinant PTN was purchased from PeproTech, Inc. (Rocky Hill, NJ, USA). Cell culture reagents were from Biochrom GmbH (Berlin, Germany). B3 peptide (CYDMKTTC) was from Cambridge Peptides (Birmingham, UK), the CDK inhibitors roscovitine and NU2058 from Santa Cruz Biotechnology, PP1, wortmannin and U0126 from TOCRIS (Minneapolis, MN, USA), and SU6656 and NSC23766 from EMD-Millipore.

### Cells and cell lines

Human endothelial cells HUVEC, human glioma U87MG cells and rat glioma C6 cells were cultured as previously described^2^. Cultures were maintained at 37 °C, 5% CO_2_, and 100% humidity.

### Mass Spectrometry

Three mg of total protein from cell lysates were immunoprecipitated with an antibody for RPTPβ/ζ and subjected to reduction with dithiothreitol and alkylation with iodoacetamide. After SDS-PAGE and silver staining of the proteins, bands were excised and treated for in-gel digestion as described^53^. Briefly, the silver was destained and trypsin (porcine, modified, sequence grade, Promega Corporation, Madison, WI, USA) was introduced to the dried gel pieces. After overnight tryptic digestion, peptides were bound to a C18 µZipTip and after washing, they were eluted with acetonitrile containing the matrix (alfa-cyano 4-hydroxycinnamic acid) directly onto the target plate. The mass list was generated by MALDI-TOF mass spectrometry on an Ultraflex TOF/TOF from Bruker Daltonics, Bremen, Germany. The search for identity was performed using the search engine ProFound (http://prowl.rockefeller.edu/prowl-cgi/ProFound). The spectrum was internally calibrated using autolytic tryptic peptides, and the error was set at +/− 0.02 Da. One missed cleavage was allowed, and methionine could be oxidized. The significance of the identity was judged from the search engines scoring system. The occurrence of the few missed cuts was either on a terminal basic residue or surrounded by acidic amino acid residues.

### Transwell migration assays

Migration assays were performed in 24-well transwell filter plates (Costar), as previously described^2^. Briefly, harvested serum-starved cells were suspended in serum free medium supplemented with 0.25% bovine serum albumin (BSA). The bottom chamber was filled with 0.6 ml of serum-free medium supplemented with 0.25% BSA and the tested substances. The upper chamber was loaded with 0.1 ml of serum-free medium containing 10^5^ cells and the chambers were incubated for 4 h at 37 °C. After completion of the incubation, the filters were fixed and stained with 0.33% toluidine blue solution. Migrating cells were quantified by counting the entire area of each filter under an Optech microscope, using a grid.

### RNA interference

HUVEC and U87MG cells were grown to a confluence of 50% and transfection was performed in fetal bovine serum (FBS)-containing medium without antibiotics for 4 h using 50 nM of annealed RNA for CDK5 (#sc-29263, Santa Cruz Biotechnology, Inc) or RPTPβ/ζ^1^ and jetSI-ENDO (Polyplus Transfection, Illkirch, France) as the transfection reagent. C6 cells were grown to a confluence of 60% and transfection was performed in serum-free medium without antibiotics for 6 h, using 80 nM annealed RNA for RPTPβ/ζ and siRNA transfection reagent (#sc-29528, Santa Cruz Biotechnology, Inc). In all cases, cells were incubated for 48 h following transfection in FBS-containing medium and serum-starved before further experiments. Double-stranded negative control siRNA (#4635, Ambion, Austin, TX) was used in all experiments.

### CDK5 kinase assays

CDK5 kinase assays were performed as previously described^54^. Briefly, cell lysates were harvested at defined time points following PTN stimulation, in the presence or absence of inhibitors, and were immunoprecipitated for CDK5. Agarose beads were washed 3 times with the lysis buffer and 2 times with a kinase reaction buffer containing proteinase and phosphatase inhibitors (20 mM Hepes, pH 7.4, 10 mM MgCl_2_, 0.5 mM EGTA, 10 mM DTT, 50 mM NaF, 50 mM β-glycerophosphate, 10 μg/ml aprotinin). Bead-bound kinase was then incubated in 20 μl of kinase buffer (20 mM Hepes, pH 7.4, 10 mM MgCl_2_, 0.5 mM EGTA, 10 mM DTT, 20 μM ATP) containing 2 μg of purified Histone H1 (#14–155, EMD-Millipore) and 20 μCi [γ-^32^P]ATP (BLU002A, PerkinElmer), at 30°C for 30 min. The reaction was terminated by adding sample loading buffer and boiling for 5 min. Protein samples were electrophoresed in SDS-PAGE and transferred to PVDF membranes. Substrate phosphorylation was analyzed by autoradiography.

Alternatively, a non-radioactive CDK5 activity assay was developed by using the ADP-Glo™ Kinase Assay kit (Promega Corporation) and 100 μg of total cell or tissue lysates immunoprecipitated with an antibody for CDK5.

### Immunoprecipitation assay

Cells were washed twice with ice-cold PBS and lysed with PBS containing 1% Triton X-100, 0.1% SDS, 20 nM sodium orthovanadate, 1 μg/ml aprotinin, 1 mM PMSF and 5 mM EDTA (lysis buffer). Cells were scraped off the plate, kept on ice for 30 min, and centrifuged at 20,000 *g* for 30 min at 4 °C. The chorioallantoic membrane (CAM) from embryos of Leghorn fertilized eggs (Pindos, Ioannina, Greece) at different developmental stages, was excised from the eggs, washed three times in PBS, homogenized in lysis buffer and centrifuged at 20,000 *g* for 30 min at 4 °C. Each experiment contained 4–5 eggs per data point. Equal total protein amounts of the supernatants from cells or CAMs were transferred to new Eppendorf tubes and incubated with primary antibodies for CDK5 (#sc-173, Santa Cruz Biotechnology), p35 (#sc-31102, Santa Cruz Biotechnology), phosphorylated tyrosine (#sc-508, Santa Cruz Biotechnology) or RPTPβ/ζ (#sc-1110, Santa Cruz Biotechnology), for 16 h at 4 °C under continuous agitation. IgG (#I2511, Sigma-Aldrich) was used as a negative control. Protein A- and protein G-agarose beads (#IP-02 and IP-04, EMD-Millipore) were added, and samples were further incubated for 2 h at 4 °C. Beads and bound proteins were collected by centrifugation and washed twice with ice-cold PBS. The pellet was resuspended with 50 μl SDS loading buffer, heated to 95–100 °C for 5 min, and centrifuged. The supernatant was analysed by Western blot analysis.

### Western blotting

Proteins were analysed by SDS-PAGE and transferred to PVDF membranes (EMD-Millipore). Blocking was performed by incubating membranes with Tris-buffered saline pH 7.4 containing Tween 20 (TBS-T), with 5% w/v nonfat dry milk for 1 h at room temperature. Membranes were further incubated in primary antibodies overnight at 4 °C under continuous agitation, as follows: mouse anti-RPTPβ/ζ (1:500 in TBS-T; #610180, BD Biosciences, San Diego, CA, USA), rabbit anti-CDK5 (1:1,000 in TBS-T; #sc-173, Santa Cruz Biotechnology), goat anti-p35 (1:1,000 in TBS-T; #sc-31102, Santa Cruz Biotechnology), β-catenin (1:1,000 in TBS-T; #9562, Cell Signaling Technology), p-integrin β_3_ (Tyr747) and integrin β_3_ (1:1,000 in TBS-T, #sc-20234 and sc-6627, Santa Cruz Biotechnology), phospho-p44/42 MAPK and p44/p42 MAPK (1:1,000 in TBS-T, #9101 and 9102, Cell Signaling Technology) and α-tubulin (1:1,000 in TBS-T, #3873, Cell Signaling Technology). Membranes were further washed three times with TBS-T and incubated in the corresponding HRP-conjugated secondary antibodies (Cell Signaling Technology or Santa Cruz Biotechnology) for 1 h at room temperature under continuous agitation. Membranes were washed three times with TBS-T and twice with TBS. Detection of immunoreactive bands was performed using the ChemiLucent^TM^ plus Western Blot enhancing kit (#2650, EMD-Millipore), according to the manufacturer’s instructions. The pictures of the gels were digitized and the protein levels that corresponded to each immunoreactive band were quantified using the ImagePC image analysis software (Scion Corporation, Frederick, MD).

### *In situ* PLA Assay

For detection of protein-protein interactions, *in situ* PLA was performed. The components used (Duolink PLA Technology, Sigma-Aldrich) were as follows: anti-mouse PLA plus probe, anti-rabbit PLA minus probe, anti-goat PLA minus probe and Detection Reagents Orange. HUVEC were grown on chamber slides (Ibidi^®^ μ-Chamber 12 well on glass slides, Martinsried, Germany) till they reached 80% confluence. Following fixation and blocking, the cells were incubated with the primary antibodies: mouse anti-RPTPβ/ζ (1:250, #610180, BD Biosciences), rabbit anti-CDK5 (1:100 in TBS-T; #sc-173, Santa Cruz Biotechnology), mouse anti-CDK5 (1:100 in TBS-T; #H00001020-M01A, Abnova, Taipei, Taiwan), goat anti-p35 (1:100 in TBS-T; #sc-31102, Santa Cruz Biotechnology). Subsequently, the cells were incubated with secondary antibodies conjugated with oligonucleotides, after hybridization and ligation of which, the DNA was amplified resulting in red fluorescence signals. Nuclei were counterstained with Draq5; cells were mounted with Mowiol 4–88 and visualized with Leica SP5 confocal microscope. Estimation of nuclei and cytoplasm size was performed using the Duolink ImageTool software. To calculate the total number of spots per cell, an algorithmic procedure was used as previously described^55^.

### Statistical analysis

The significance of variability between the results from each group and the corresponding controls was determined by unpaired t-test or ANOVA, as appropriate. Each experiment included triplicate measurements for each condition tested, unless otherwise indicated.

### Data availability

All data generated or analysed during this study are included in this published article and its Supplementary information file.

## Electronic supplementary material

Dataset 1
