# Supplementary material for: Cyclin-dependent kinase 5 mediates pleiotrophin-induced endothelial cell migration
Source: Sci Rep. 2018 Apr 12;8:5893. doi: 10.1038/s41598-018-24326-x (PMC5897396; doi:10.1038/s41598-018-24326-x)

Cyclin-dependent kinase 5 mediates pleiotrophin-induced endothelial cell migration

Evgenia Lampropoulou[1], Ioanna Logoviti[1], Marina Koutsioumpa[1,#], Maria Hatziapostolou[2], Christos Polytarchou[2], Spyros S. Skandalis[3,4], Ulf Hellman[4], Manolis Fousteris[5], Sotirios Nikolaropoulos[5], Efrosini Choleva[1], Margarita Lamprou[1], Angeliki Skoura[6], Vasileios Megalooikonomou[6] and Evangelia Papadimitriou[1,*]

[1]Laboratory of Molecular Pharmacology, Department of Pharmacy, University of Patras, GR26504 Patras, Greece; [2]Department of Biosciences, School of Science and Technology, Nottingham Trent University, Nottingham, NG11 8NS, United Kingdom; [3]Laboratory of Biochemistry, Department of Chemistry, University of Patras, GR26504 Patras, Greece; [4]Ludwig Institute for Cancer Research, Uppsala University, Uppsala SE-751-05, Sweden; [5]Laboratory of Medicinal Chemistry, Department of Pharmacy, University of Patras, GR26504 Patras, Greece; [6]Computer Engineering and Informatics Department, University of Patras, Patras, Greece.

[#]Current address: Center for Systems Biomedicine, Vatche and Tamar Manoukian Division of Digestive Diseases, David Geffen School of Medicine, University of California at Los Angeles, Los Angeles, CA, 90095, USA

[*]Author of correspondence: E. Papadimitriou, Ph.D., Laboratory of Molecular Pharmacology, Department of Pharmacy, University of Patras, GR 26504 Patras, Greece. Tel/FAX: 0030-2610-962336

e-mail: epapad@upatras.gr

Supplementary Figure S1

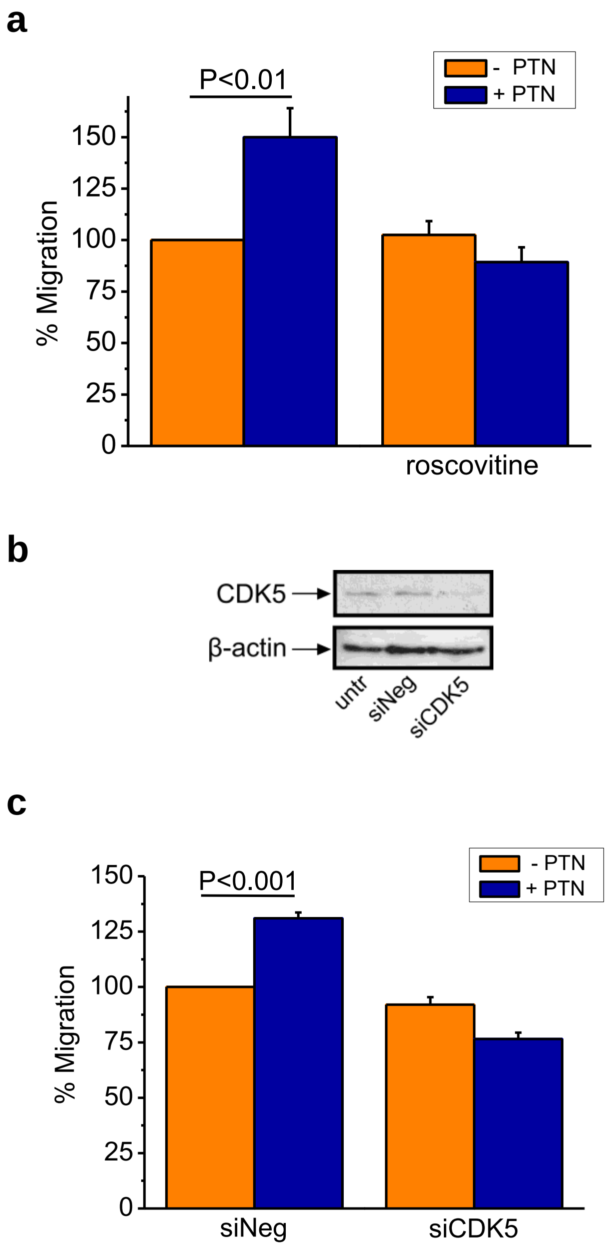

CDK5 is involved in PTN-induced cell migration. (a) Serum-starved human glioma U87MG cells were stimulated by PTN (100 ng/ml) in the absence or presence of roscovitine (10 μM). Migration was measured using the transwell assay, as described in Materials and Methods. Results are expressed as mean ± s.e.m. (n=4) of the percentage change in the number of migrated cells compared with the untreated cells (set as default 100%). **(b)** Representative picture from Western blot analysis of total U87MG cell lysates following down-regulation of CDK5 by siRNA. **(c)** Following down-regulation of CDK5, serum-starved U87MG cells were stimulated by PTN and migration was measured using the transwell assay. Results are expressed as mean ± s.e.m. (n=3) of the percentage change in the number of migrated cells compared with the untreated siNeg cells (set as default 100%). siNeg, cells transfected with a negative control siRNA; siCDK5, cells transfected with siRNA for CDK5. F values of the ANOVA tests are 9.9 for (a) and 79.9 for (c).

Supplementary Figure S2

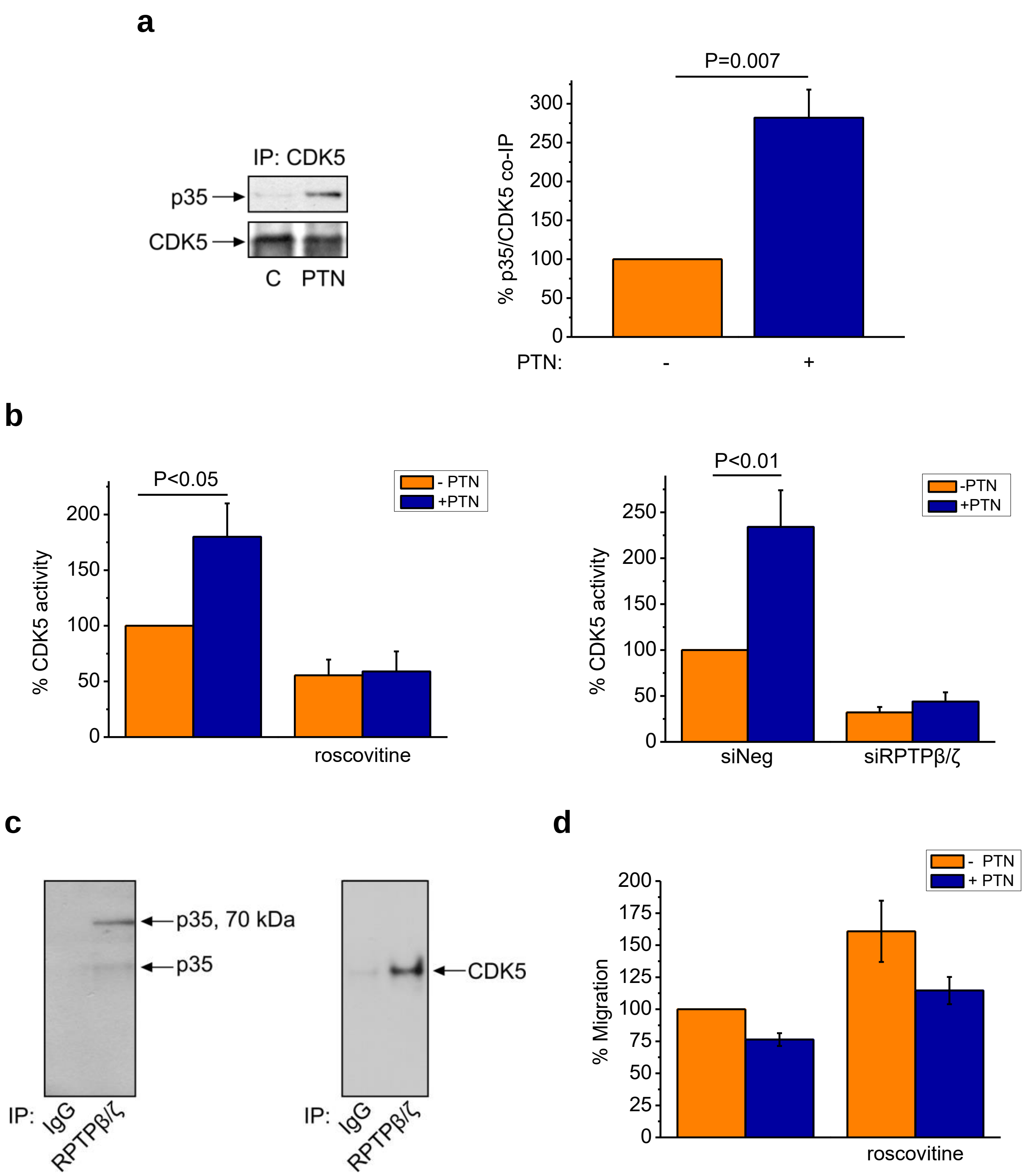

PTN-induced CDK5 activation depends on RPTPβ/ζ but not $\alpha_v\beta_3$ integrin. Rat glioma C6 cells that do not express $\alpha_v\beta_3$[2] were incubated with PTN (100 ng/ml) in the absence or presence of roscovitine (10 μM) or after down-regulation of RPTPβ/ζ expression by siRNA. **(a)** Total cell lysates were immunoprecipitated with an antibody for CDK5 and the immunoprecipitates were analyzed by Western blot for the presence of CDK5 and p35. CDK5 and p35 protein amounts were quantified and the ratio of p35 to CDK5 was calculated in each lane. Results are expressed as mean ± s.e.m. (n=3) of the percent change in p35 to CDK5 relative amounts in PTN-stimulated vs. untreated C6 cells (set as default 100%). **(b)** CDK5 activity was measured in CDK5 immunoprecipitates from C6 cells by using the ADP-Glo Kinase Assay as described in Materials and Methods. Results are expressed as mean ± s.e.m. (n≥3) of the percent change in CDK5 activity compared with the untreated cells (set as default 100%). siNeg, cells transfected with a negative control siRNA; siCDK5, cells transfected with siRNA for CDK5. **(c)** Total cell lysates from C6 cells were immunoprecipitated with an antibody for RPTPβ/ζ and analyzed by

Western blot for the presence of p35 and CDK5. Pictures are representative from three independent experiments. **(d)** Serum-starved C6 cells were stimulated by PTN in the absence or presence of roscovitine. Migration was measured using the transwell assay, as described in Materials and Methods. Results are expressed as mean ± s.e.m. (n=4) of the percentage change in the number of migrated cells compared with the untreated cells (set as default 100%). F values of the ANOVA tests are 7.2 (left) and 19 (right) for (b) and 7.2 for (d).

Supplementary Figure S3

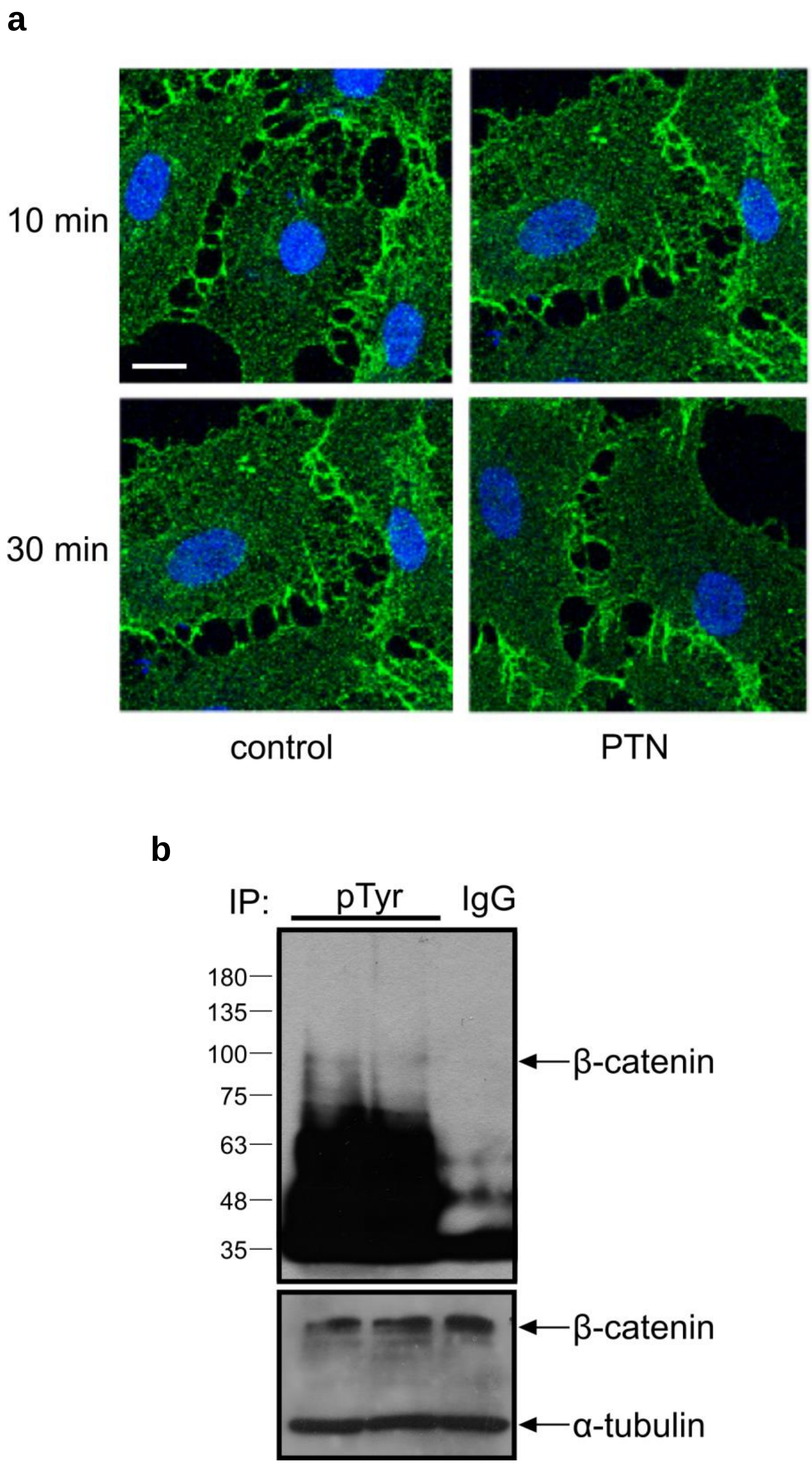

PTN does not affect cellular localization or tyrosine phosphorylation of β-catenin in HUVEC. (a) Serum-starved HUVEC were stimulated by PTN (100 ng/ml) for 10 and 30 min. Cells were fixed with 3.7% paraformaldehyde in phosphate-buffered saline (PBS), pH 7.4, for 10 min and permeabilized with PBS containing 0.1% Triton. After being washed 3 times with PBS, the cells were blocked with PBS containing 3% bovine serum albumin (BSA) and 10% fetal bovine serum (FBS) for 1 h at room

temperature. The cells were stained with a primary antibody against β-catenin and an Alexa secondary antibody (#A-21441, Molecular Probes, Carlsbad, CA), both used at the concentration of 1:500. Nuclei were stained with Draq5 and the cells were mounted with Mowiol 4-88 and visualized at 21°C with Leica SP5 (X63 objective with a numerical aperture of 1.4) confocal microscope. The figure shows representative immunofluorescence images of HUVEC stained for β-catenin (green) and nucleus (blue). Scale bar corresponds to 10 μm. **(b)** Serum-starved HUVEC were stimulated by PTN (100 ng/ml) for 10 min. Total HUVEC lysates were immunoprecipitated with an antibody for phosphorylated tyrosine (pTyr) and analyzed by Western blot for the presence of β-catenin (upper blot). The same total cell lysates were immunoblotted using antibodies for β-catenin and α-tubulin as loading controls (lower blot). Pictures in both cases are representative from two independent experiments.

Supplementary Figure S4

a

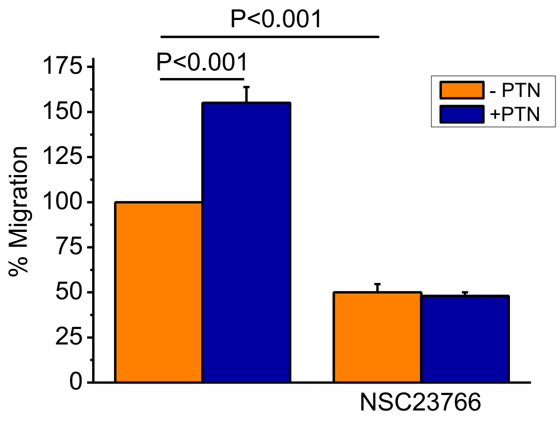

b

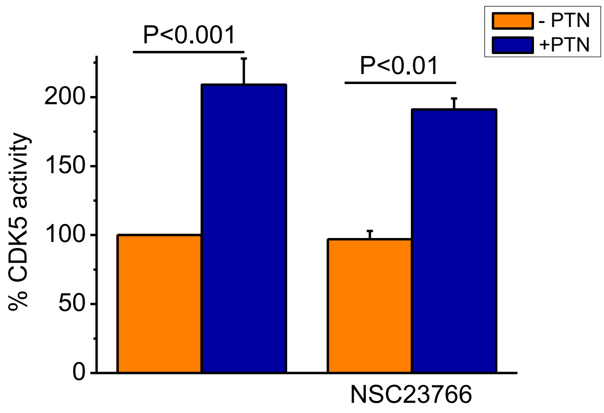

Involvement of Rac1 in PTN-induced HUVEC migration. (a) Serum-starved HUVEC were stimulated by PTN (100 ng/ml) in the absence or presence of the selective Rac1 inhibitor NSC23766 (50 μM). Migration was measured using the transwell assay, as described in Materials and Methods. Results are expressed as mean ± s.e.m. (n=3) of the percentage change in the number of migrated cells compared with the untreated cells (set as default 100%). **(b)** CDK5 activity was measured in CDK5 immunoprecipitates from HUVEC by using the ADP-Glo Kinase Assay as described in Materials and Methods. Results are expressed as mean ± s.e.m. (n=3) of the percent change in CDK5 activity compared with the untreated cells (set as default 100%). F values of the ANOVA tests are 99.8 for (a) and 31.3 for (b).

Supplementary Figure S5

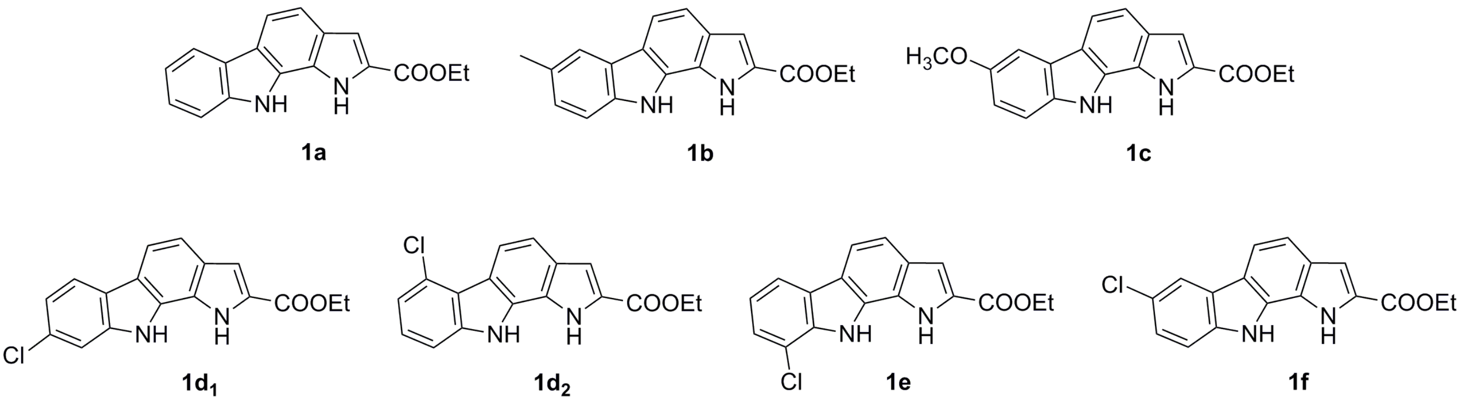

Chemical structures of the pyrrolo[2,3-*α*]carbazole derivatives studied[17,18].

Supplementary Figure S6

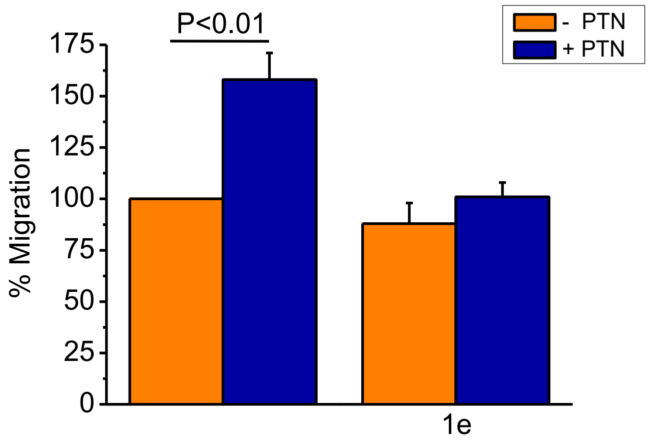

Effect of the pyrrolo[2,3-*α*]carbazole derivative 1e on PTN-induced migration of human glioma U87MG cells *in vitro*. Serum-starved U87MG cells were stimulated by PTN (100 ng/ml) in the absence or presence of the 1e derivative (10 µM). Migration was measured using the transwell assay, as described in Materials and Methods. Results are expressed as mean ± s.e.m. (n=3) of the percentage change in the number of migrated cells compared with the untreated cells (set as default 100%). F value of the ANOVA test is 12.4.

Supplementary Figure S7

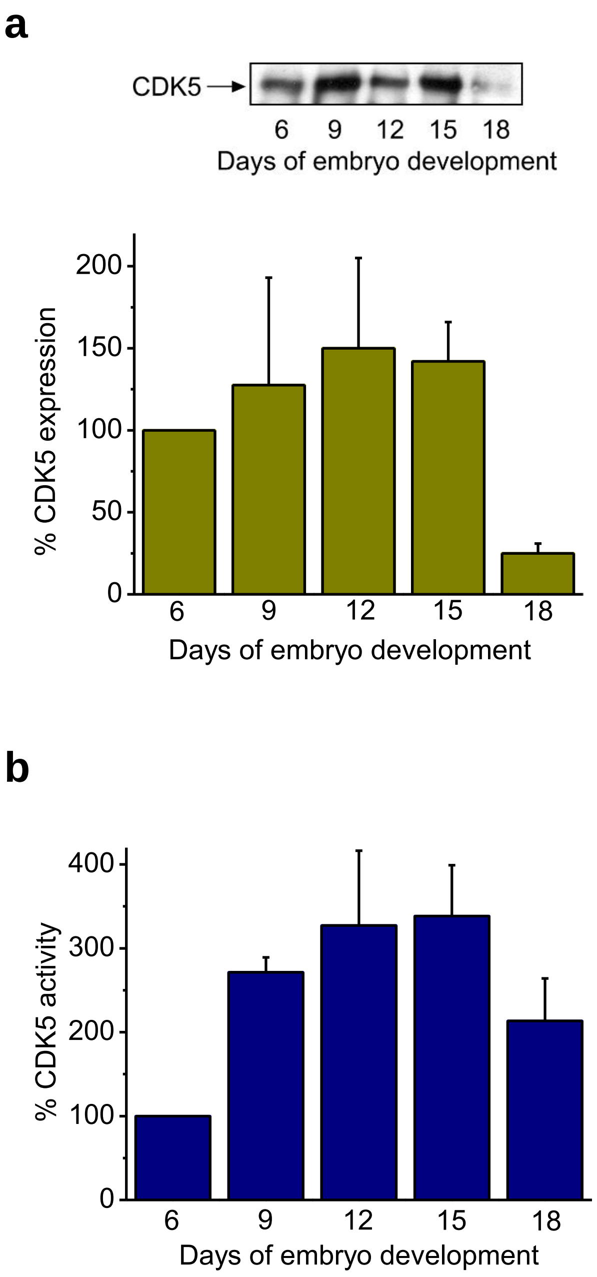

CDK5 expression (a) and activity (b) during development in the chicken embryo CAM. Equal amounts of total protein CAM extracts (500 μg) derived from different developmental stages were immunoprecipitated with a CDK5 antibody and immunoprecipitates were analysed for CDK5 protein amounts by Western blot analysis **(a)** and for CDK5 activity by using the ADP-Glo Kinase Assay **(b)**. Results are expressed as mean ± s.e.m. of the percent CDK expression (n=2) or activity (n=3) compared to the corresponding levels at day 6 of embryo development (set as default 100%). F values of the ANOVA tests are 1.6 for (a) and 3.3 for (b).

Blots that appear cropped in the manuscript

Figure 2b

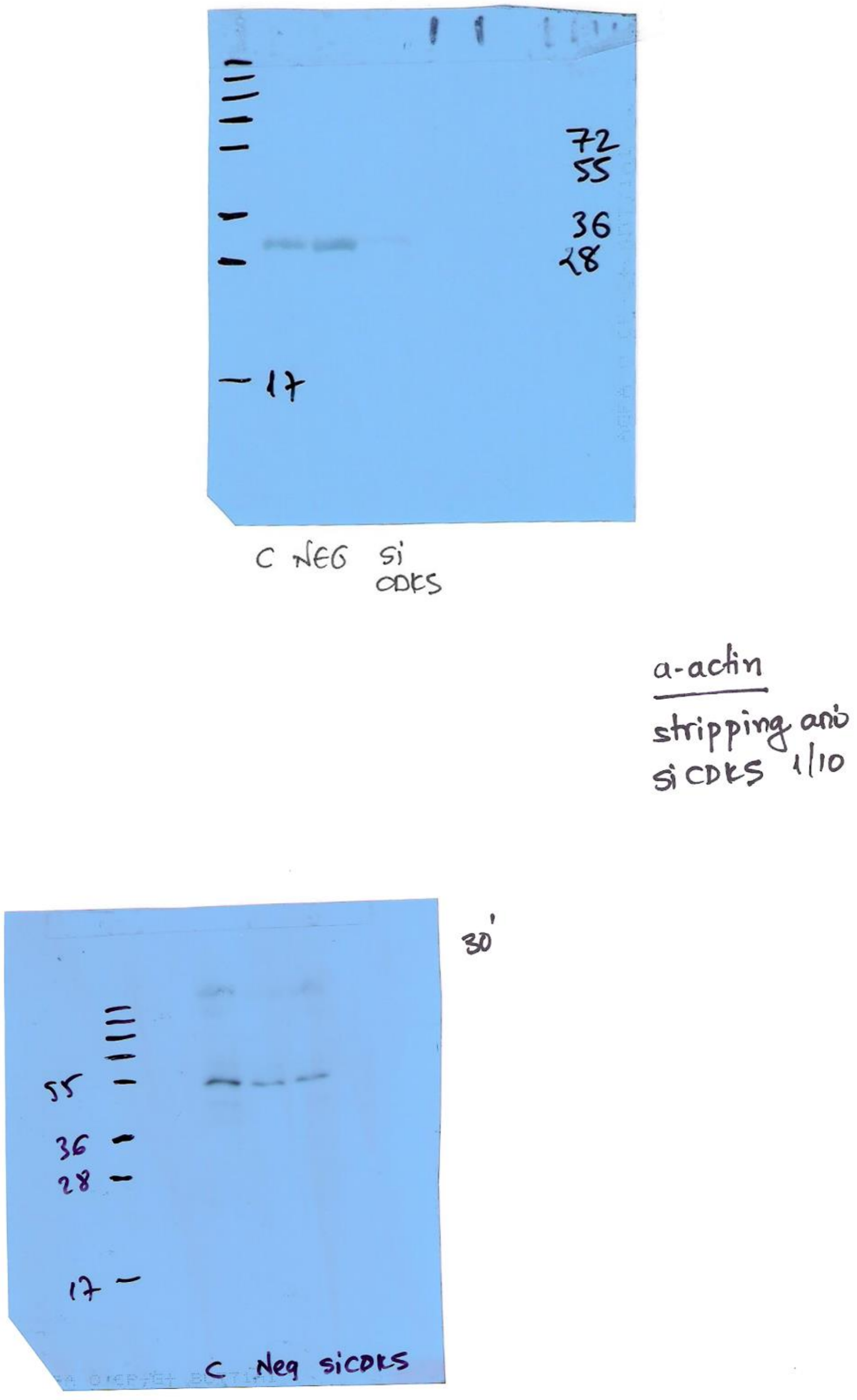
72
55
36
28
17
C NEG si CDK5
a-actin
stripping anti siCDK5 1/10
30'
55
36
28
17
C Neg siCDK5

Figure 3a

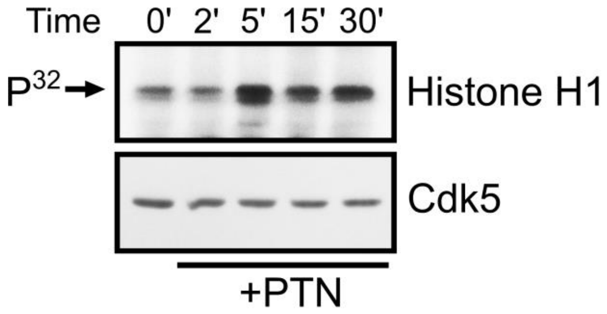

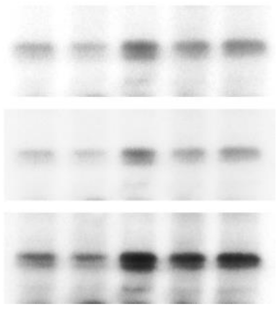

Figure 3b

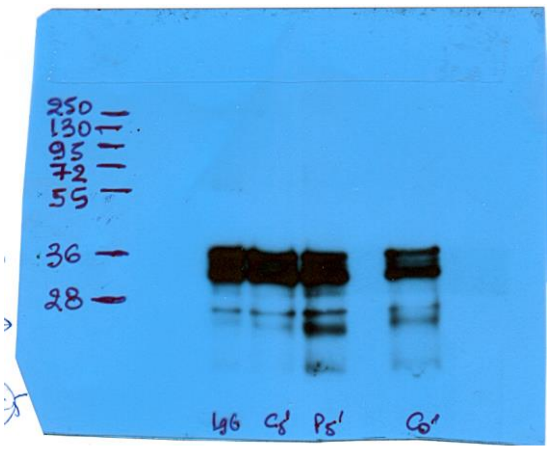

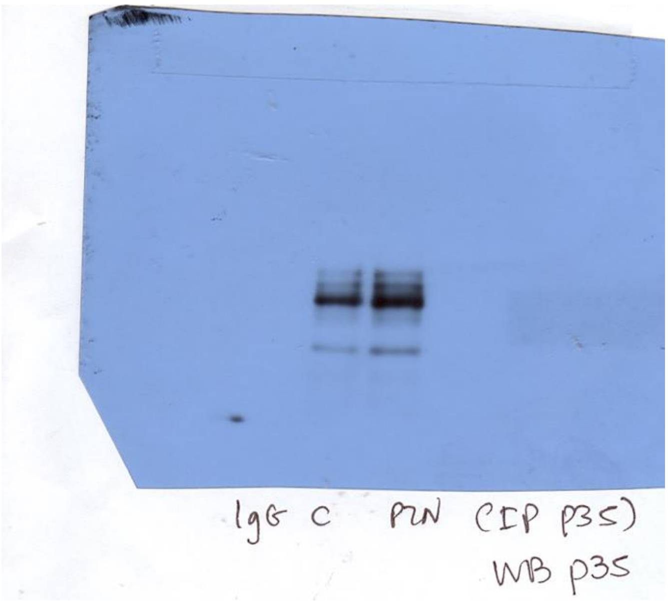

Figure 4b

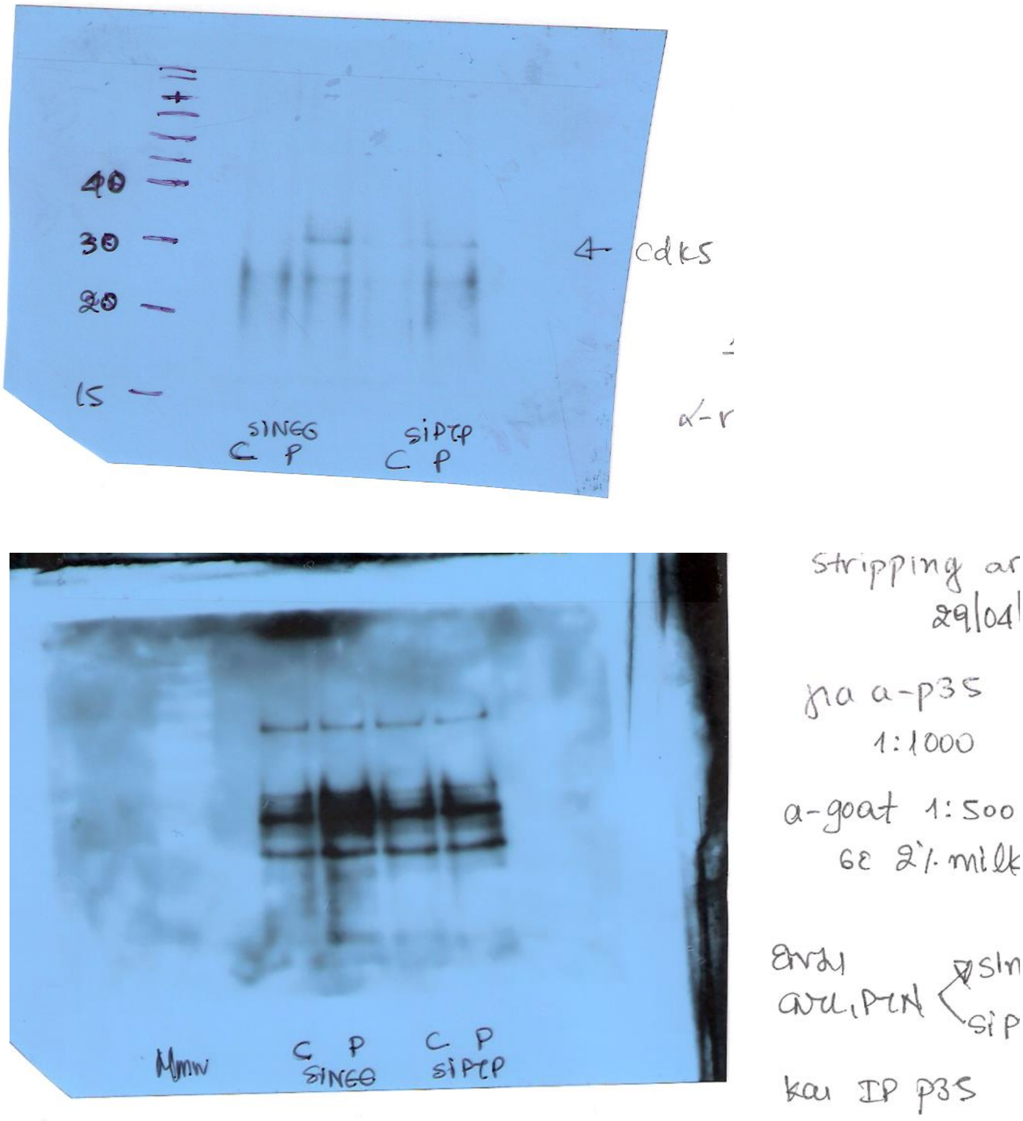
40
30
20
15
SINEG
C P
siPTP
C P
cdk5
Stripping
29/04
a-p35
1:1000
a-goat 1:500
2% milk
Mmw
C P
SINEG
C P
siPTP
IP p35

Figure 4c

CDK5

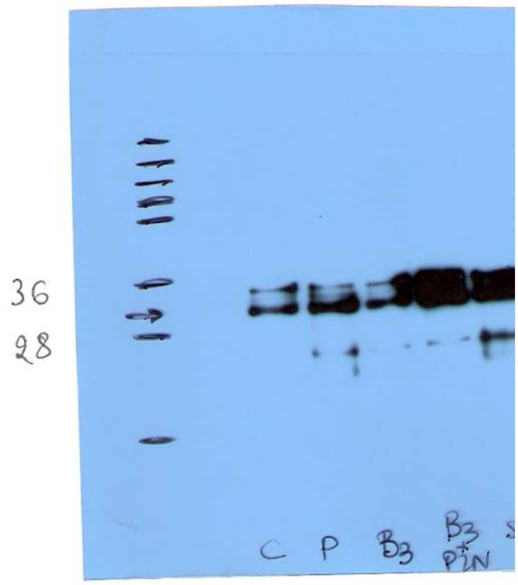

p35

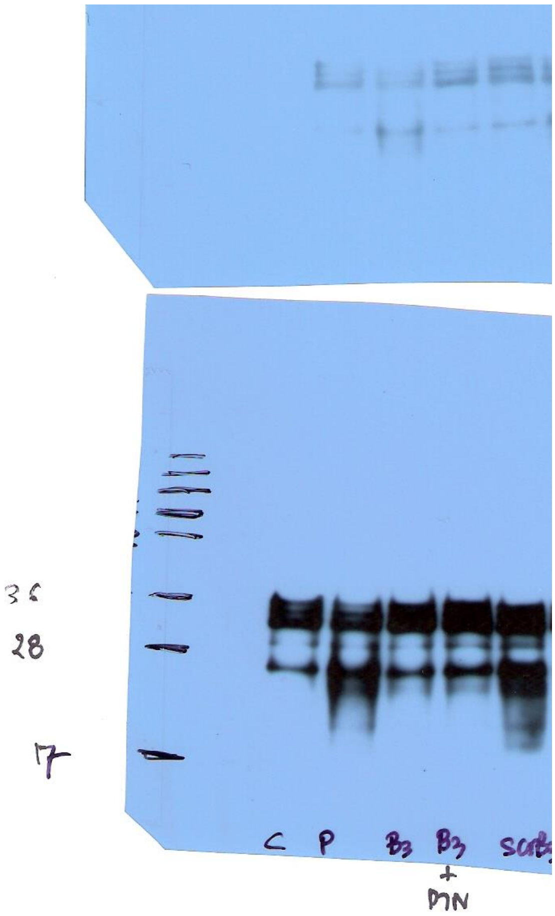

Figure 4d

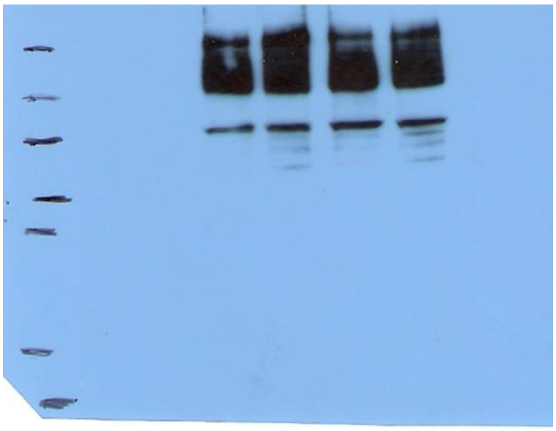

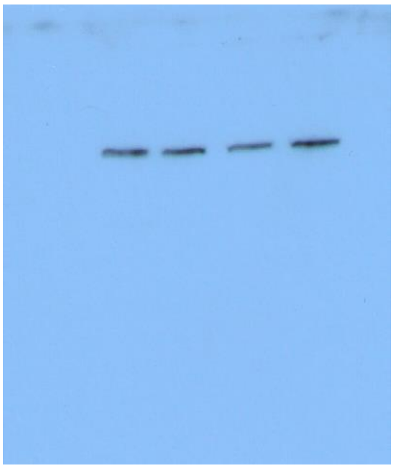

Figure 5b

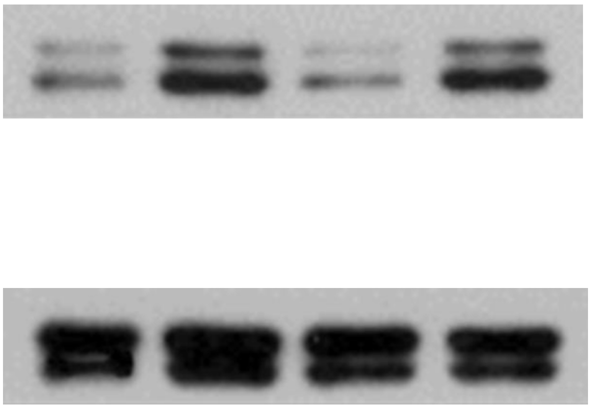

Figure 6a

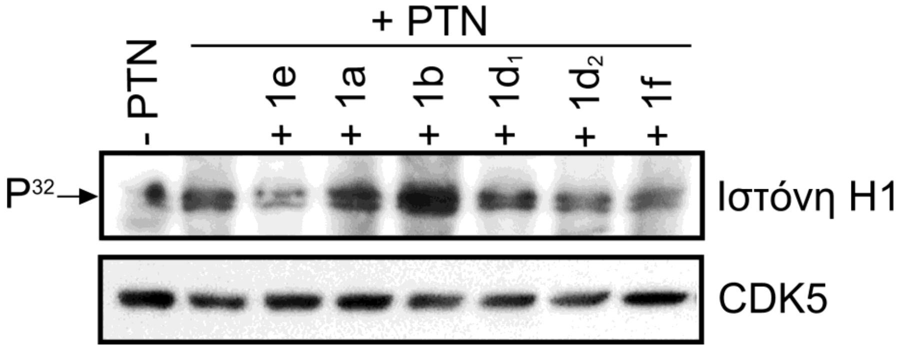

Supplementary Figure S1

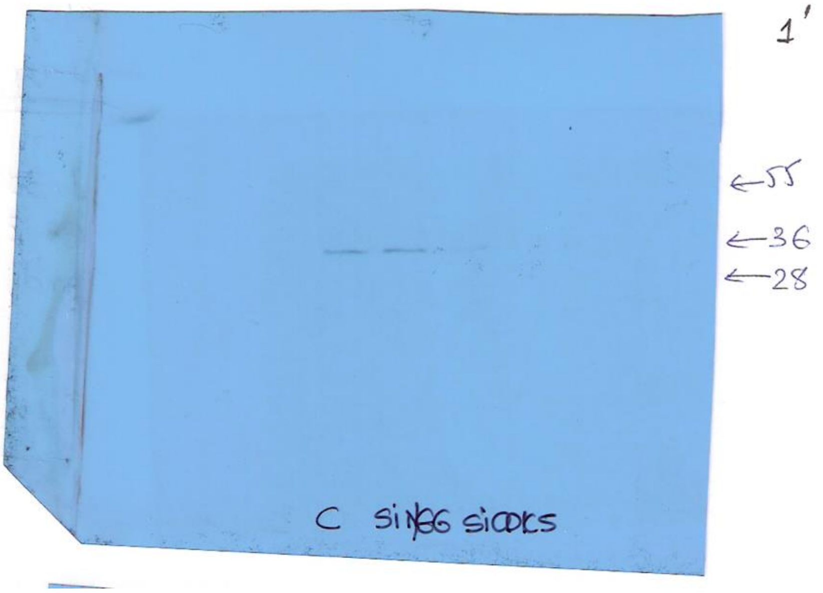

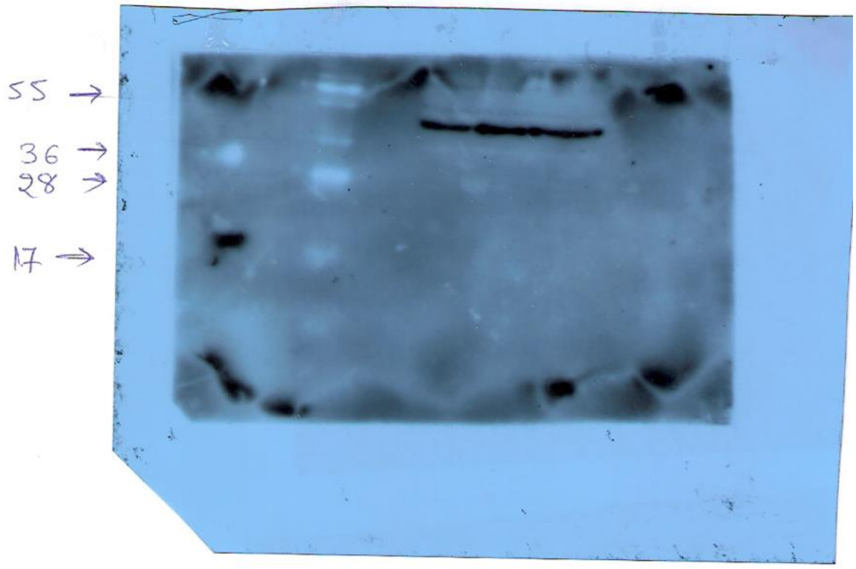

Supplementary Figure S2a

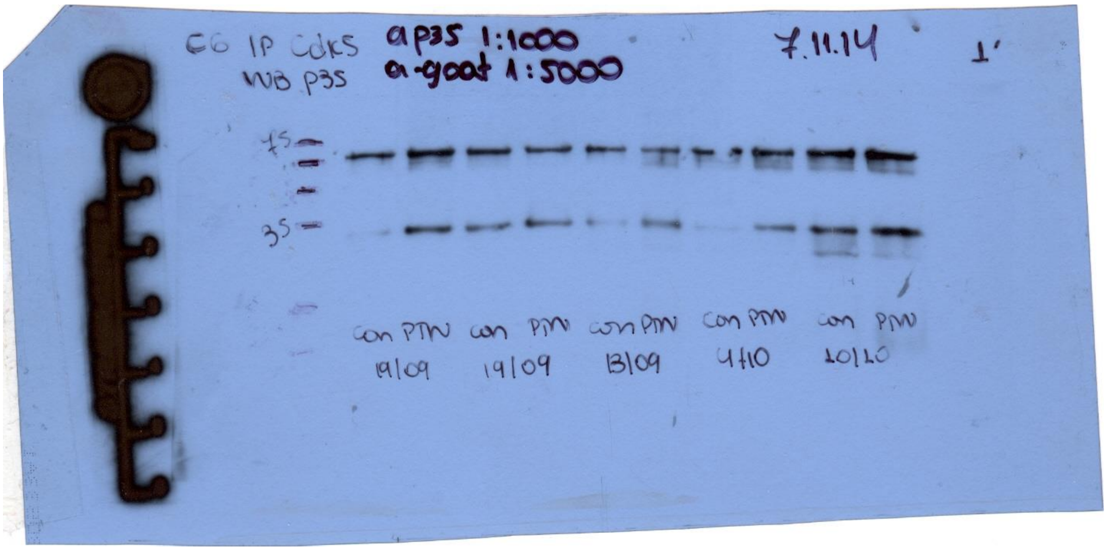

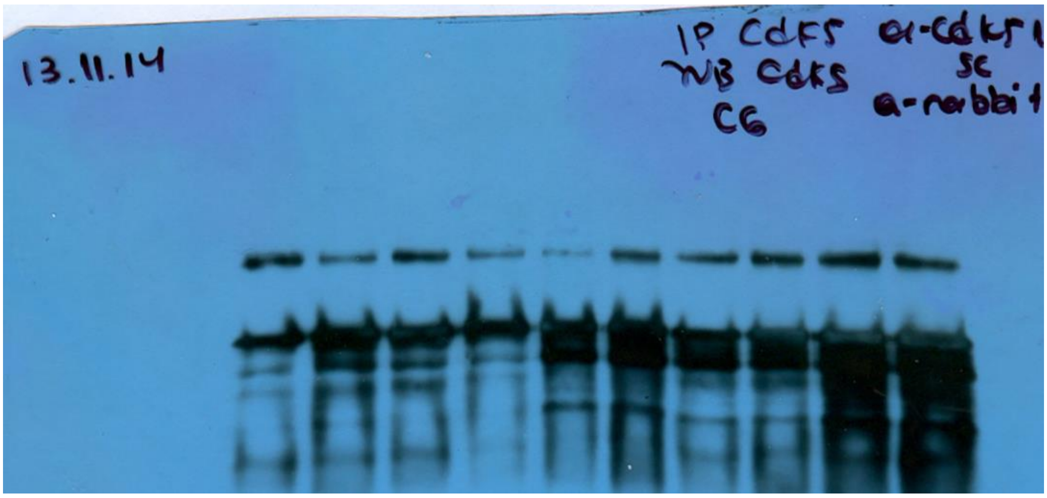

Supplementary Figure S7a

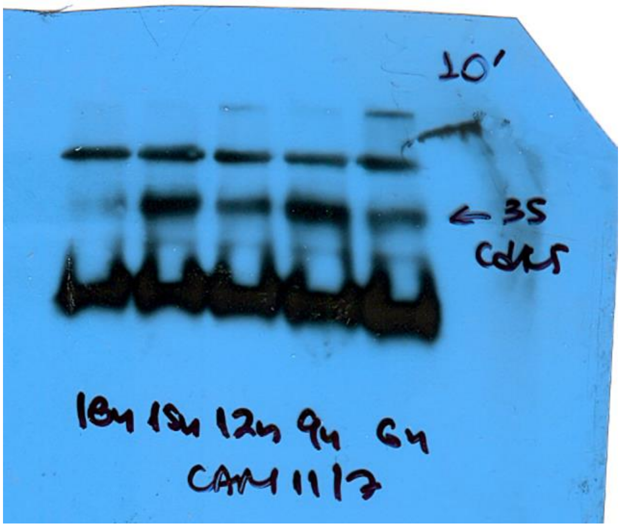

Supplement: Supplementary file 1 — Dataset 1 [file 41598_2018_24326_MOESM1_ESM.pdf]
